# Computational Exploration on the Structural and Optical Properties of Gold-Doped Alkaline-Earth Magnesium AuMg_n_ (*n* = 2–12) Nanoclusters: DFT Study

**DOI:** 10.3389/fchem.2022.870985

**Published:** 2022-03-29

**Authors:** Ben-Chao Zhu, Ping-Ji Deng, Jia Guo, Wen-Bin Kang

**Affiliations:** School of Public Health, Hubei University of Medicine, Shiyan, China

**Keywords:** calypso, DFT, AuMgn nanoclusters, optical properties, structural property

## Abstract

Using CALYPSO crystal search software, the structural growth mechanism, relative stability, charge transfer, chemical bonding and optical properties of AuMg_n_ (*n* = 2–12) nanoclusters were extensively investigated based on DFT. The shape development uncovers two interesting properties of AuMg_n_ nanoclusters contrasted with other doped Mg-based clusters, in particular, the planar design of AuMg_3_ and the highly symmetrical cage-like of AuMg_9_. The relative stability study shows that AuMg_10_ has the robust local stability, followed by AuMg_9_. In all nanoclusters, the charge is transferred from the Mg atoms to the Au atoms. Chemical bonding properties were confirmed by ELF analysis that Mg-Mg formed covalent bonds in nanoclusters larger than AuMg_3_. Static polarizability and hyperpolarizability calculations strongly suggest that AuMg_9_ nanocluster possesses interesting nonlinear optical properties. Boltzmann distribution weighted average IR and Raman spectroscopy studies at room temperature verify that these nanoclusters are identifiable by spectroscopic experiments. Finally, the average bond distance and average nearest neighbor distance were fully investigated.

## Introduction

Metal nanoclusters have attracted increasing interest from academics in recent years due to their appealing micro patterns and interesting features (Jin et al., 2016; Peng et al., 2018; Tew et al., 2018). For example, Au_n_ clusters tend to exhibit 2-dimensional structures at small sizes, while medium sizes (*n* < 15) will transition to 3-dimension (Idrobo et al., 2007; Assadollahzadeh and Schwerdtfeger, 2009; Huang and Wang, 2009). For larger size, the study of Au_144_ cluster is highly worth explaining. It was first reported in 1997 as a critical size for the transformation of Au nanoclusters into nanocrystals and worthy of being researched, but its structure could not be determined at that time (Alvarez et al., 1997), then in 2009 it was precisely predicted by theoretical studies to have a multishell structure (Qian and Jin, 2009), and finally, in 2018 it was experimentally confirmed to have a three-layer metallic core of Au_12_-Au_42_-Au_60_ from the inside out (Yan et al., 2018). Researchers have been so persistent in studying them because the physical size of these clusters is comparable to the electron Fermi wavelength and therefore tends to show interesting electronic (Yau et al., 2013), optical properties (Ramakrishna et al., 2008; Jin, 2015) and have important application prospects in the field of medicine and biology (Shang et al., 2011). Because the physical and chemical characteristics of nanoclusters alter with size throughout the transition to nanocrystals or nanoparticles, nanocluster research will anticipate, at least theoretically, a slew of new materials for the field of nanomaterials science.

A lot of studies on alkaline Earth metal magnesium clusters have been reported, in addition to usual studies of metal nanoclusters like gold, silver, and copper (Köhn et al., 2001; Xia et al., 2016; Zhang et al., 2020; Zhao et al., 2021). This is partly because magnesium-based nanomaterials have an exceptional hydrogen storage capacity compared to ordinary materials (Shao et al., 2012), and therefore, various kinds of Mg-based nanocluster materials, such as CoMg_n_ (Trivedi and Bandyopadhyay, 2015), RhMg_n_ (Trivedi and Bandyopadhyay, 2016), ScMg nanocluster (Chen et al., 2020; Lyon, 2021), are worthy of systematic study. Most of these studies were carried out theoretically and gave interesting results by predicting the hydrogen storage properties of nanocluster materials based on Mg. For example, it is shown that MgScH_13_ and MgScH_15_ nanoclusters have, theoretically, ultra-high hydrogen storage capacities of 15.9 wt% (Lyon, 2021) and 17.8 wt% (Chen et al., 2020), respectively. On the other hand, the optical properties of Mg and Mg-based nanoclusters are also very attractive (Belyaev et al., 2016; Shinde, 2016; Shinde and Shukla, 2017). The IR spectra of Mg_n_ (*n* = 2–31) nanoclusters were studied using DFT, and the results showed that their most intense peaks were distributed in the low-frequency band of 40–270 cm^−1^ (Belyaev et al., 2016). The linear absorption spectroscopy studies of very small size Mg_n_ (*n* = 2–5) nanoclusters confirm that their low-lying structures, although small, can be experimentally distinguished, while the optical excitation spectra are confirmed to be of plasmonic collective type (Shinde, 2016).

In short, nanocluster materials, like Au and Mg, are a field of materials science full of unknown “surprises” where many interesting optical and electronic excitation properties can be “discovered”. However, surprisingly, the study of gold-doped Mg nanoclusters has not been reported so far. This work aims to perform a systematic theoretical computational study of the structural and optical properties of gold-doped small-size magnesium nanoclusters. Specifically, the geometric growth mechanism, relative stability, charge transfer properties, chemical bonding properties, nonlinear optical properties, and theoretical calculations of infrared and Raman-weighted average spectra of AuMg_n_ (*n* = 2–12) nanoclusters will be investigated. These studies will not only enrich the research data on AuMg_n_ nanoclusters, but also provide the opportunity to gain insight into potential nanomaterials with interesting optical properties.

## Computational Method Details

CALYPSO software (Wang et al., 2010, 2012) was utilized to search the initial geometries of AuMg_n_ (*n* = 2–12) nanoclusters. CALYPSO can perform predictions of the energetically low-lying isomers structures at given chemical compositions and pressure for nanoclusters (Lv et al., 2012; Zhao et al., 2019; Lu et al., 2020) in gas-phase and crystals (Lu and Chen, 2020a, 2020b, 2021; Sun et al., 2020; Chen et al., 2021) *via* particle swarm optimization (PSO) algorithm. To search for as many low-lying energy isomers of AuMg_n_ (*n* = 2–12) nanoclusters as possible, the following strategy will be employed. First, each size of AuMgn nanocluster will be searched for 50 generations, where each generation contains 20 structures. Further, 80% of these 1,000 heterogeneous structures are generated by the PSO algorithm for the initial structure, and the rest are generated randomly. These structures are then interfaced *via* CALYPSO to Gaussian 09 software (Frisch et al., 2016) for low-level HF energy calculations, and finally ranked by energy level. It is necessary to explain that many of the 1,000 isomers obtained have the same or extremely close energies, and their geometrical structures do not differ much and therefore need to be removed. Finally, isomers with significantly different energies and structures were again subjected to high-level DFT for structural optimization and frequency calculations by Gaussian 09 software. The structure optimization calculation employs the classical B3LYP functional, where the mixed basis set is considered due to the presence of Au atom. Concretely, 6–311 g (d) is applied to Mg atoms, while the pseudopotential basis set lanl2dz is used for Au atoms. The adoption of such functional and basis set is based on the following two aspects, firstly, the existing studies have shown that Mg_n_ nanoclusters do not have any metallic properties at the size of *n* < 20 (Köhn et al., 2001; Jellinek and Acioli, 2003; Xia et al., 2016), and secondly, all-electron basis set and lanl2dz basis set have been proved to be reliable for Mg and Au nanoclusters by numerous studies (Xia et al., 2016; Zeng et al., 2020; Zhu et al., 2021). To ensure a more comprehensive structural optimization, each isomer was calculated under 2, 4, 6, and 8 spin multiplicities, respectively. In addition, to verify that the isomer is not a transition or excited state, imaginary frequencies must be excluded from any result. Once the imaginary frequency appears in the calculation result, they need to be optimized again until all frequencies are positive.

Charge transfer property of the lowest energy AuMg_n_ (*n* = 2–12) nanoclusters was analyzed by natural bond orbital (NBO) calculation (Reed et al., 1988). Chemical bonding properties were computed through the electron localization function (ELF) (Becke and Edgecombe, 1990). The nonlinear optical properties of the ground state AuMg_n_ nanoclusters were investigated at the aug-cc-pVTZ level. Infrared and Raman spectra are the results of vibration frequency calculations. In particular, Multiwfn software (Lu and Chen, 2012) is a powerful tool to draw 2D map of ELF, spherical plots of static and super-static polarizabilities, Boltzmann distribution probabilities of different isomers and weighted average IR and Raman spectral data.

## Results

### The Geometrical Growth Mechanism of AuMg_n_ Nanoclusters

**FIGURE 1:**
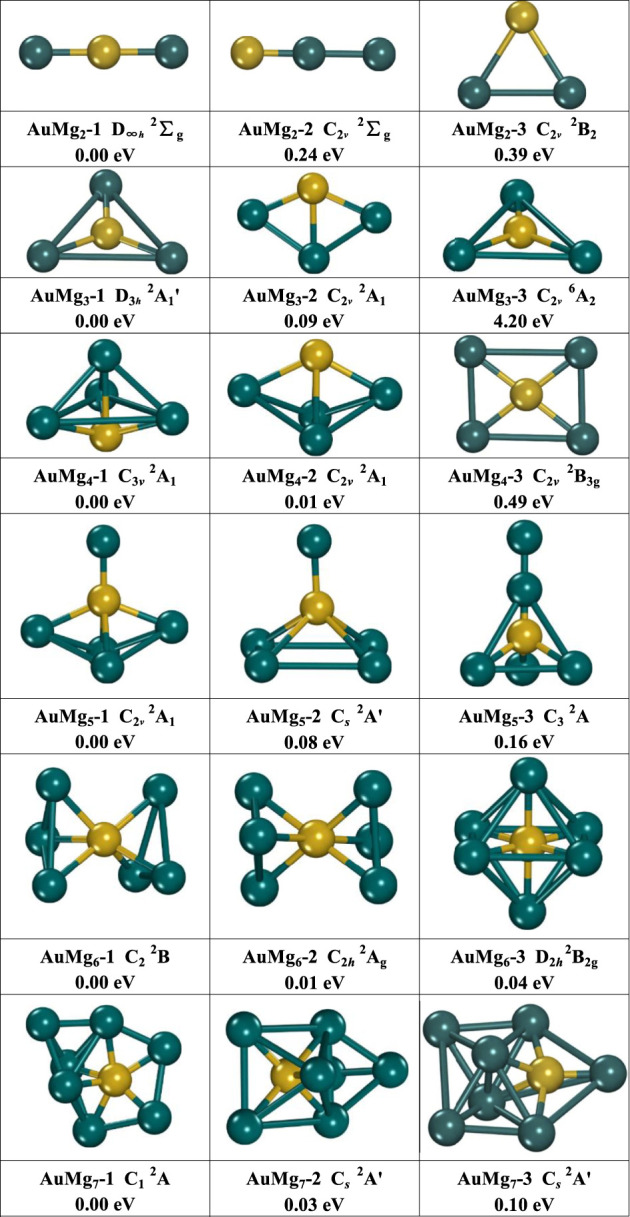
The geometry of the three lowest energy isomers of AuMg_n_ (*n* = 2–7) nanoclusters, energy difference in eV from the lowest energy isomer at the same size.

**FIGURE 2:**
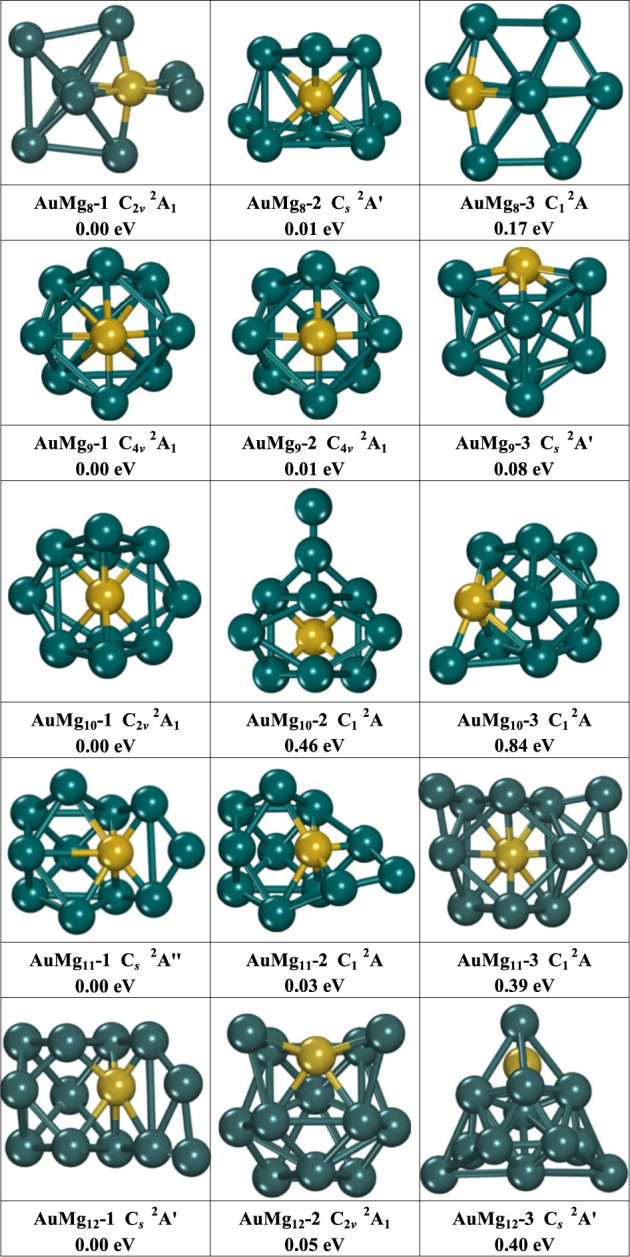
The geometry of the three lowest energy isomers of AuMg_n_ (*n* = 8–12) nanoclusters, energy difference in eV from the lowest energy isomer at the same size.

The growth mechanism of nanoclusters can be studied by their geometric structures. Three low-lying energy isomers of each size AuMg_n_ (*n* = 2–12) nanoclusters are presented in Figure 1 and Figure 2. Under each structure, the “i” in AuMg_n_-i is their energy order, with “1” indicating the lowest and “2” the second-lowest ones. The energy difference (eV) between the AuMgn-i and AuMg_n_-1 at each size can also be found. In addition to the symmetry, and the electronic structure information is also shown in Figures 1, 2. All information about the lowest energy state of AuMg_n_ (*n* = 2–12) nanoclusters is summarized in Table 1, where the results of the frequency calculations show the lowest and highest vibrational frequencies satisfying the requirements that the results of the frequency calculations cannot contain any imaginary frequency. As can be seen from Figure 1, isomers AuMg_2_-1 (D_∞*h*_ symmetry with ^2^Σ_g_ electronic state) and AuMg_2_-2 (C_2*v*_ symmetry with ^2^Σ_g_ electronic state) have a similar linear structure, the difference being that the Au atom is in the center of the former and on the side of the latter. Isomers AuMg_2_-3 (C_2*v*_ symmetry with ^2^B_2_ electronic state) show a 2D planar isosceles triangle structure. Relative to the lowest energy state AuMg_2_-1 isomer energy, AuMg_2_-2 and AuMg_2_-3 isomers have 0.24 and 0.39 eV higher energy than it, respectively. The isomer AuMg_3_-1 (D_3*h*_ symmetry with ^2^Aʹ_1_ electronic state) has an equilateral triangular geometry, while the isomer AuMg_3_-3 (C_2*v*_ symmetry with ^6^A_2_ electronic state) has an isosceles triangular structure, and interestingly the Au atoms are located at the center of their triangular structures, respectively. The structure of the isomer AuMg_3_-2 (C_2*v*_ symmetry with ^2^A_1_ electronic state) is a planar combination of Au-Mg-Mg isosceles triangle and Mg-Mg-Mg isosceles triangle. Calculations show nanoclusters of 2 Mg atoms doped with one Au atom, where the second and third lowest isomers are 0.03 and 4.20 eV higher than the lowest energy isomer, respectively. The structures of the isomers AuMg_4_-1 (C_3*v*_ symmetry with ^2^A_1_ electronic state) and AuMg_4_-2 (C_2*v*_ symmetry with ^2^A_1_ electronic state) can be considered as formed based on the tetrahedral (pyramid-like) structure of Au-Mg-Mg-Mg adsorbing an Mg atom in different directions. The isomer AuMg_4_-3 (C_2*v*_ symmetry with ^2^B_3g_ electronic state) shows a rectangular structure in which the 4 Mg atoms are at the vertices while Au atom is at the geometric center. For the isomer AuMg_4_-1, AuMg_4_-2 and AuMg_4_-3 are higher in energy by 0.01 and 0.49 eV, respectively. The structure of the isomer AuMg_5_-1 (C_2*v*_ symmetry with ^2^A_1_ electronic state) is based on the formation of AuMg_4_-2 by adsorbing an Mg atom on its top. The isomers AuMg_4_-3 and AuMg_4_-1 form the isomers AuMg_5_-2 (C_*s*_ symmetry with ^2^Aʹ electronic state) and AuMg_5_-3 (C_3_ symmetry with ^2^A electronic state) after pulling up the Au atom into the interior of the polyhedral while adsorbing an Mg atom on their tops. The second- and third-lowest energy isomers of the AuMg_5_ nanocluster are 0.08 and 0.16 eV higher than that of the lowest-energy isomer. The isomers AuMg_6_-1 (C_2_ symmetry with ^2^B electronic state) and AuMg_6_-2 (C_2*h*_ symmetry with ^2^A_g_ electronic state) have extremely close energies and structures, which exhibit rotational symmetry with Au atom. The isomer AuMg_6_-3 (^2^B_2g_ electronic state), which is higher 0.04 eV in energy than AuMg_6_-1, possesses a high symmetry (D_2*h*_) octahedron in which the Au atom is located at its center. The structures of the isomers AuMg_7_-1 (C_1_ symmetry with ^2^A electronic state), AuMg_7_-2 (C_*s*_ symmetry with ^2^Aʹ electronic state) and AuMg_7_-3 (C_*s*_ symmetry with ^2^Aʹ electronic state) are all grown based on the diversity of Au-Mg-Mg-Mg tetrahedra-like geometries. In addition, the energy shift of the isomers AuMg_7_-2 and AuMg_7_-3 relative to the lowest energy state are 0.03 and 0.10 eV, respectively.

**TABLE 1:** State, symmetry, Eb, ∆_2_E, Egap for α- and β-electrons, frequency and NCP on Au atom in the ground state of AuMg_n_ (*n* = 2–12) nanoclusters.

Clusters	State	Symmetry	E_b_(eV)	∆_2_E(eV)	E_gap_α(eV)	E_gap_β(eV)	Frequency (cm^−1^)		NCP on Au (e)
							Highest	Lowest	
AuMg_2_	D_∞*h*_	^2^∑_g_	1.72	—	2.76	2.53	220	14	−0.74
AuMg_3_	D_3*h*_	^2^A_1_′	1.78	−0.15	2.28	2.22	197	25	−1.35
AuMg_4_	C_3*v*_	^2^A_1_	1.84	0.29	2.19	2.15	212	23	−1.68
AuMg_5_	C_2*v*_	^2^A_1_	1.83	−0.03	2.28	1.29	232	13	−2.17
AuMg_6_	C_2_	^2^B	1.83	−0.04	2.21	1.14	225	9	−2.99
AuMg_7_	C_1_	^2^A	1.84	−0.05	1.74	1.65	207	10	−2.44
AuMg_8_	C_2*v*_	^2^A_1_	1.85	−0.32	1.55	1.58	203	20	−3.05
AuMg_9_	C_4*v*_	^2^A_1_	1.89	0.24	1.63	1.75	236	53	−1.00
AuMg_10_	C_2*v*_	^2^A_1_	1.90	0.28	1.14	1.85	224	21	−2.49
AuMg_11_	C_*s*_	^2^A″	1.89	−0.12	1.70	1.39	232	27	−2.39
AuMg_12_	C_*s*_	^2^A′	1.88	—	1.15	1.88	243	13	−2.47

As Figure 2 displayed, the medium-sized AuMg_n_ (8–12) nanoclusters exhibit a diversity of structures. The structures of the isomers AuMg_8_-1 (C_2*v*_ symmetry with ^2^A_1_ electronic state) and AuMg_8_-3 (C_1_ symmetry with ^2^A electronic state) are generated based on the deformation of AuMg_7_-3 after adsorption of an Mg atom, while the structure of the isomer AuMg_8_-2 (C_*s*_ symmetry with ^2^Aʹ electronic state) can be obtained from the deformation of AuMg_7_-2 by adsorption of an Mg atom. The second and third lowest energy isomers of AuMg_8_ nanoclusters are higher in energy than the ground state by 0.01 and 0.17 eV. The isomers AuMg_9_-1 and AuMg_9_-2 have the same symmetry (C_4*v*_), electronic structure (^2^A_1_), energy and “fascinating cage-like” structures. The isomer AuMg_9_-3 (^2^Aʹ electronic state), which is 0.08 eV higher in energy than AuMg_9_-1, has a cage-like structure with C_*s*_ symmetry. The structures of the isomers AuMg_10_-1 (C_2*v*_ symmetry with ^2^A_1_ electronic state), AuMg_10_-2 (C_1_ symmetry with ^2^A electronic state) and AuMg_10_-3 (C_1_ symmetry with ^2^A electronic state) are generated based on the deformation of AuMg_9_-1 by adsorption of Mg atoms in different directions. The second and third lowest energy isomers of AuMg_10_ nanoclusters are higher in energy than the first lowest energy by 0.46 and 0.84 eV, respectively. Interestingly, the isomers AuMg_11_-1 (C_*s*_ symmetry with ^2^Aʹ electronic state) and AuMg_11_-2 (C_1_ symmetry with ^2^A electronic state) are easily obtained by the deformation of AuMg_10_-2 by adsorption of an Mg atom. On the other hand, AuMg_11_-3 (C_1_ symmetry with ^2^A electronic state) is formed by the deformation of AuMg10-1 after the adsorption of an Mg atom. AuMg_11_-2 and AuMg_11_-3 have higher energies than AuMg_11_-1 at 0.03 and 0.39 eV. The isomer AuMg_12_-1 (C_*s*_ symmetry with ^2^Aʹ electronic state) is generated by the adsorption of an Mg atom by AuMg_11_-1. The isomer AuMg_12_-2 (C_2*v*_ symmetry with ^2^A_1_ electronic state), on the other hand, exhibits a deformed tubular-like structure, while the isomer AuMg_12_-3 (C_*s*_ symmetry with ^2^Aʹ electronic state) has a pyramid-like structure. Furthermore, compared to the energy of AuMg_12_-1, AuMg_12_-2 and AuMg_12-_3 are 0.05 and 0.40 eV higher, respectively. Because the lowest energy state isomers of nanoclusters often require more comprehensive studies to explore their various physical and chemical properties, the atomic coordinates of the AuMg_n_-1 (*n* = 2–20) nanoclusters are shown in Supplementary Table S1 in the Supplemental Material.

In conclusion, based on the small size of AuMg_n-1_ or smaller, AuMg_n_ (*n* = 2–12) nanoclusters can usually be formed by adsorption of Mg atoms in different directions, and the interesting point is that the direction of adsorption does not have a fixed pattern. Such result is consistent with many existed Mg-based nanoclusters reported (Li et al., 2017; Zeng et al., 2020, 2021; Zhu et al., 2020). However, despite the many similarities, the structures of gold-doped Mg nanoclusters have unique properties compared to other Mg-based nanoclusters studies. For example, the structure of AuMg_3_ nanoclusters is 2D planar, while the lowest energy heterostructures of Be (Zeng et al., 2020), Si (Zhu et al., 2020), C, Ge, Sn (Zeng et al., 2021), Zn-doped (Li et al., 2017) Mg nanoclusters of corresponding sizes are all ortho-tetrahedral in shape. Interestingly, although the ground-state structures of AuMg_9_ and BeMg_9_ (Zeng et al., 2020) look similar, the significant difference between them is that the Au atom locates on the surface of AuMg_9_ while the Be atom is absorbed into the inside of BeMg_9_.

### The Relative Stabilities

Since clusters exhibit different physical and chemical properties at different sizes, their relative stability is well worth studying. The relative stability of cluster can be calculated through the following three quantities, that is the binding energy per atom (*E*_b_ in eV), the second-order energy difference (Δ_2_ *E* in eV) and the HOMO-LUMO energy gap (*E*_gap_ in eV). Equations 1–3 display the above three energies for AuMg_n_-1 (*n* = 2–20) nanoclusters in Figure 1.

$$E_{b}\left({\text{AuMg}}_{\text{n}}\right)=\left[nE\left(\text{Mg}\right)+E\left(\text{Au}\right)-E\left({\text{AuMg}}_{\text{n}}\right)\right]/\left(n+1\right)$$ (1)

$$\Delta_{2}E\left({\text{AuMg}}_{\text{n}}\right)=E\left({\text{AuMg}}_{\text{n}+1}\right)+E\left({\text{AuMg}}_{\text{n}-1}\right)-2E\left({\text{AuMg}}_{\text{n}}\right)$$ (2)

$$E_{\text{gap}}\left({\text{AuMg}}_{n}\right)=E_{\text{LUMO}}\left({\text{AuMg}}_{n}\right)-E_{\text{HOMO}}\left({\text{AuMg}}_{n}\right)$$ (3)

*E* (Au) and *E* (Mg) denote the energies of free Au and Mg atoms, *E* (AuMg_n_) means the energy of the corresponding nanocluster. The lowest unoccupied molecular orbital (LUMO) and highest occupied molecular orbital (HOMO) energies are *E*_LUMO_ and *E*_HOMO_.

**FIGURE 3:**
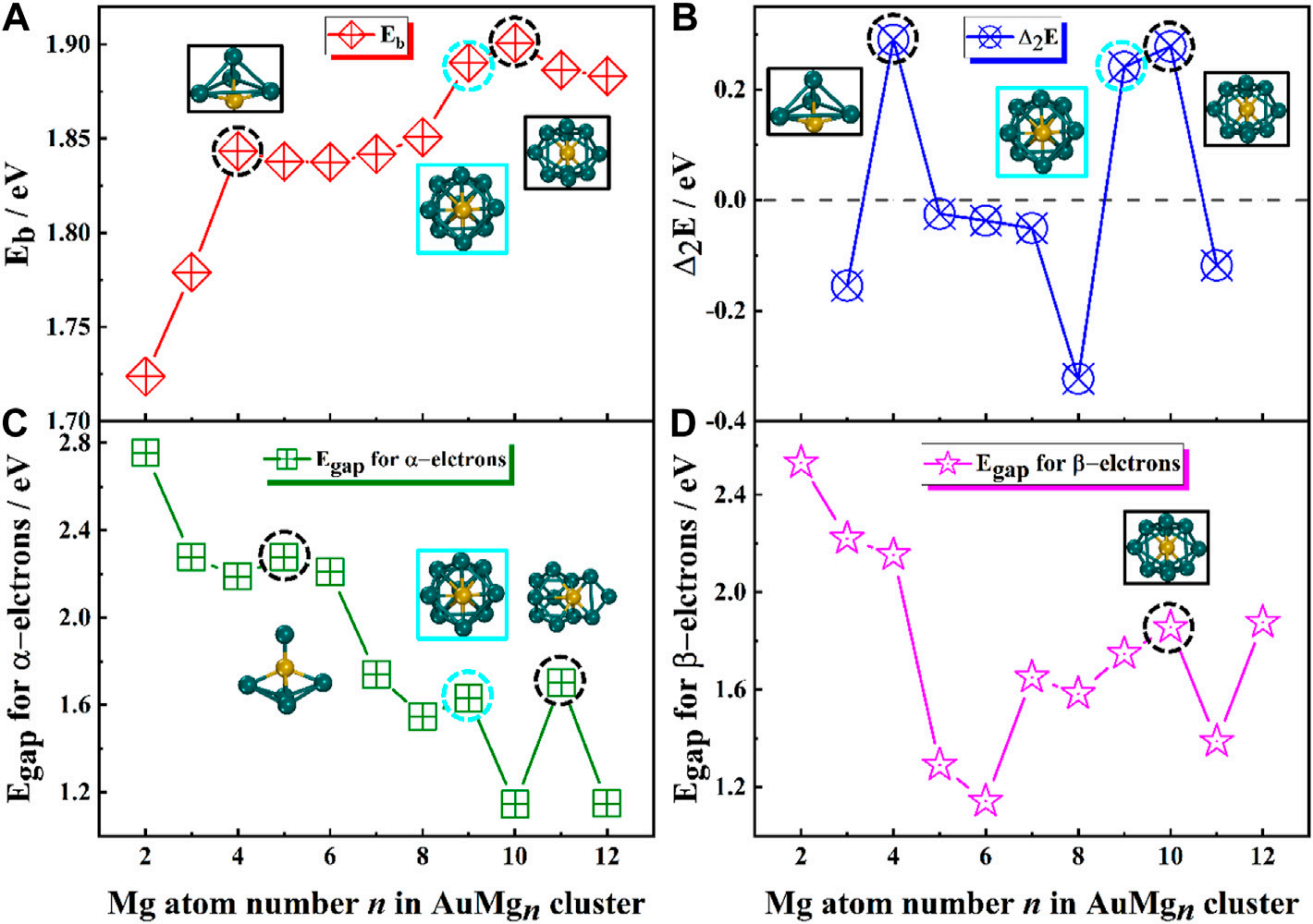
**(A)** Average binding energy E_b_, **(B)** The second order difference energy Δ_2_E, **(C)** The HOMO-LUMO energy gap E_gap_ for α-electrons, **(D)** The HOMO-LUMO energy gap E_gap_ for β-electrons in the ground state of AuMg_n_ (*n* = 2–12) nanoclusters.

The theoretically calculated values of these quantities for AuMg_n_-1 (*n* = 2–20) nanoclusters are presented in Table 1 and their curves with size are showed in Figure 3. As Figure 3A displayed, overall, the *E*_b_ curve becomes larger as the size of the nanoclusters increases, implying that the atoms within the AuMg_n_ nanoclusters bind more stably as the Au atoms are doped. Locally, the maximum value of *E*_b_ appears at AuMg_10_ (1.90 eV), indicating that this nanocluster has the robust stability. Secondly, a small local peak (1.84 eV) appears at AuMg_4_, indicating that it is slightly more stable than its neighbors. Figure 3B shows the curve of the Δ_2_E, which can be detected experimentally by mass spectrometry. Interestingly, as in the case of the local peaks of the *E*_b_ curve, AuMg_4_ and AuMg_10_ have local maximum Δ_2_E values of 0.29 and 0.28 eV, respectively. This conclusion suggests that they are both the most stable and have a high probability of being observed in mass spectrometry experiments. The thermodynamic stability of nanoclusters can be characterized by the value of their *E*_gap_. Since AuMg_n_ nanoclusters are open-shell systems, they have both α and β-electrons, and the *E*_gap_ of α and β-electrons are illustrated in Figure 3C and Figure 3D. For α-electrons *E*_gap_ curve of AuMg_n_ nanoclusters, the local peaks appear at *n* = 5, 9 and 11, while AuMg_10_ has the largest local β-electron *E*_gap_, indicating that the thermal stability of these clusters is relatively high.

In conclusion, AuMg_4_ and AuMg_10_ nanoclusters show the robust stability. However, it is noteworthy that the AuMg_9_ nanocluster is always the second largest value in both E_b_ and Δ_2_E curves, although they are not the maximum values. Therefore, combined with the *E*_gap_ maximum for α-electrons, AuMg_9_ nanocluster also has considerable robust stability and be worthy studying.

### Charge Transfer Property and Chemical Bond Analysis

**FIGURE 4:**
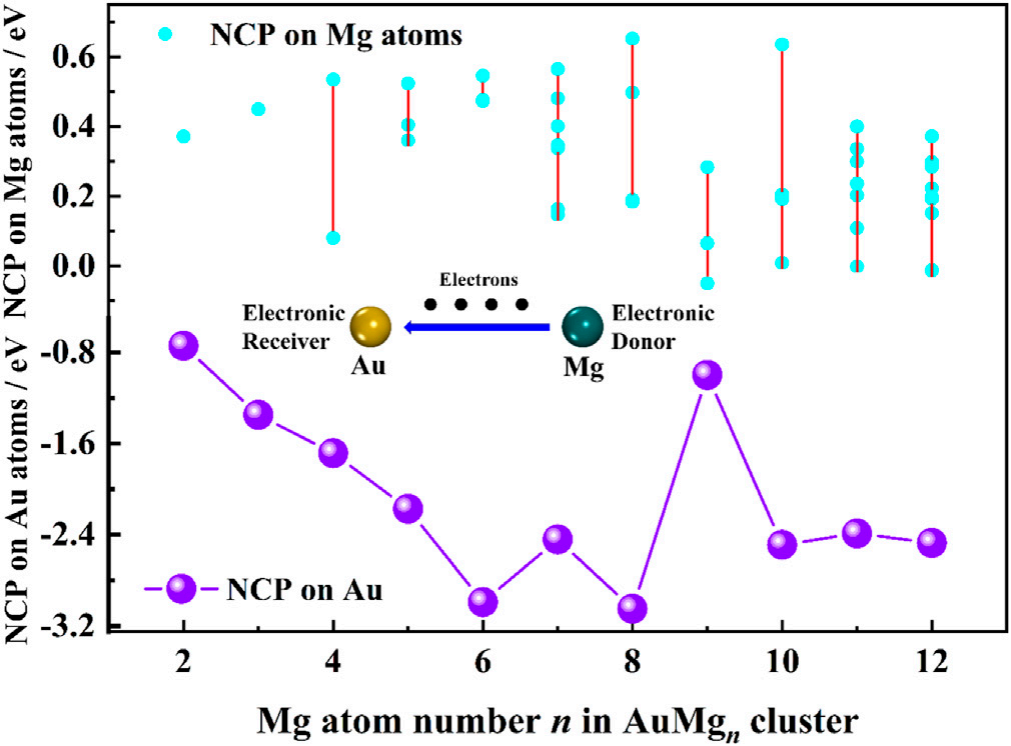
NCP analysis on Au and Mg atoms in the ground state of AuMg_n_ (*n* = 2–12) nanoclusters.

The natural charge population (NCP) results from the NBO calculations can reveal the charge transfer properties in the nanoclusters. The NCP values of Au atoms in Table 1 are in the range of [−2.99, −0.74] e, indicating that Au atoms play the role of electron receiver in all nanoclusters. The curve of NCP on Au atom with the size is ploted in Figure 4. The AuMg_9_ nanocluster appears to be very special, which is probably originated from its high symmetry structure. Supplementary Table S2 in the Supplementary Material shows the NCP values on the Mg atoms. Except for 4 Mg atoms in AuMg_9_ with a charge of −0.05 e and 1 Mg atom in AuMg_12_ with an NCP value of −0.01 e, all other Mg atoms have positive NCP values, distributed from 0.65 to 0.01 e, suggesting that they are losing electrons. In other words, Mg atoms are electron donors in the AnMg_n_ nanoclusters. The charge transfer property depends on the electronegativity of the atom, the greater the electronegativity, the easier it is to get electrons. The electronegativity value of Mg atom is 1.31, while that of Au atom is 2.54, so the charge transfer is mostly from Mg atom to Au atom.

**FIGURE 5:**
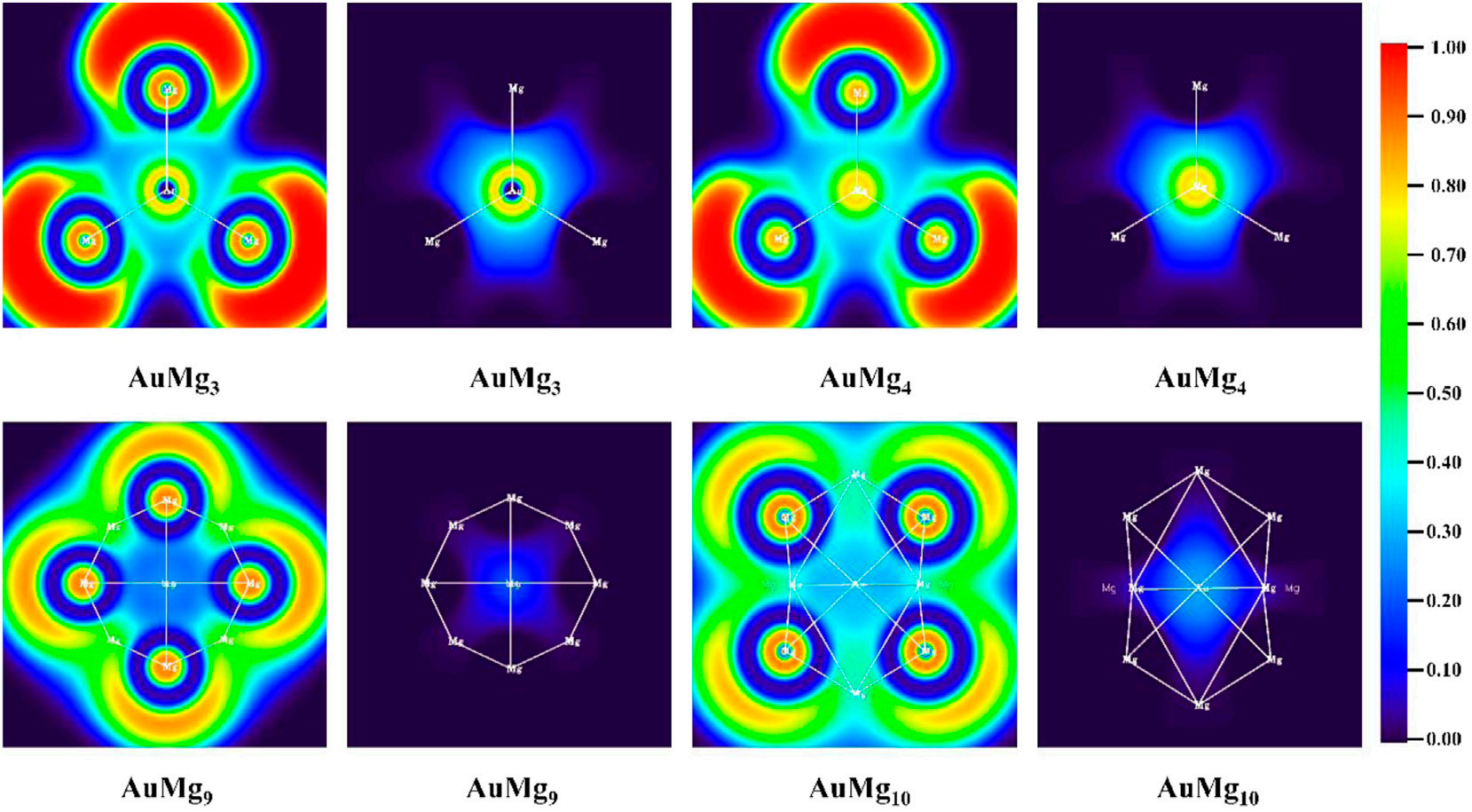
The ELF analysis for Mg-Mg and Au-Mg chemical bonds for AuMg_n_ (*n* = 3, 4, 9, 10) nanoclusters.

The ELF values of atomic bonding regions and their 2D maps are useful tools for analyzing the chemical bonding properties of nanoclusters. ELF is a value greater than 0 and less than 1, which characterizes the degree of electron localization and thus can determine the bonding properties. A region with ELF > 0.5 implies high electron localization and covalent bonding in the bonding region, while a region with ELF < 0.5 has low electron localization and non-covalent bonding in the bonding region. Figure 5 and Supplementary Figure S1 in the Supplementary Material display the 2D distribution of the EFL value for the ground state AuMg_n_ (*n* = 2–12) nanoclusters. The calculations show that Mg-Mg in AuMg_n_ nanoclusters is covalently bonded, while Au-Mg bond is non-covalent. The relatively large value of ELF around Au atom and low in bonding region indicates that the valence layer of Au atom is solidified around it, so it does not form covalent bonds with Mg. Furthermore, considering that the Au atom always gains electrons and the Mg atom around it loses electrons to be positively charged, it can be concluded that Au-Mg is ionic bonding. Another noteworthy point is that the critical size for Mg-Mg bonding is AuMg_4_. ELF distribution map shows that Mg-Mg does not covalently bond in AuMg_2_ and AuMg_3_ nanoclusters.

### The Nonlinear Optical Property

**FIGURE 6:**
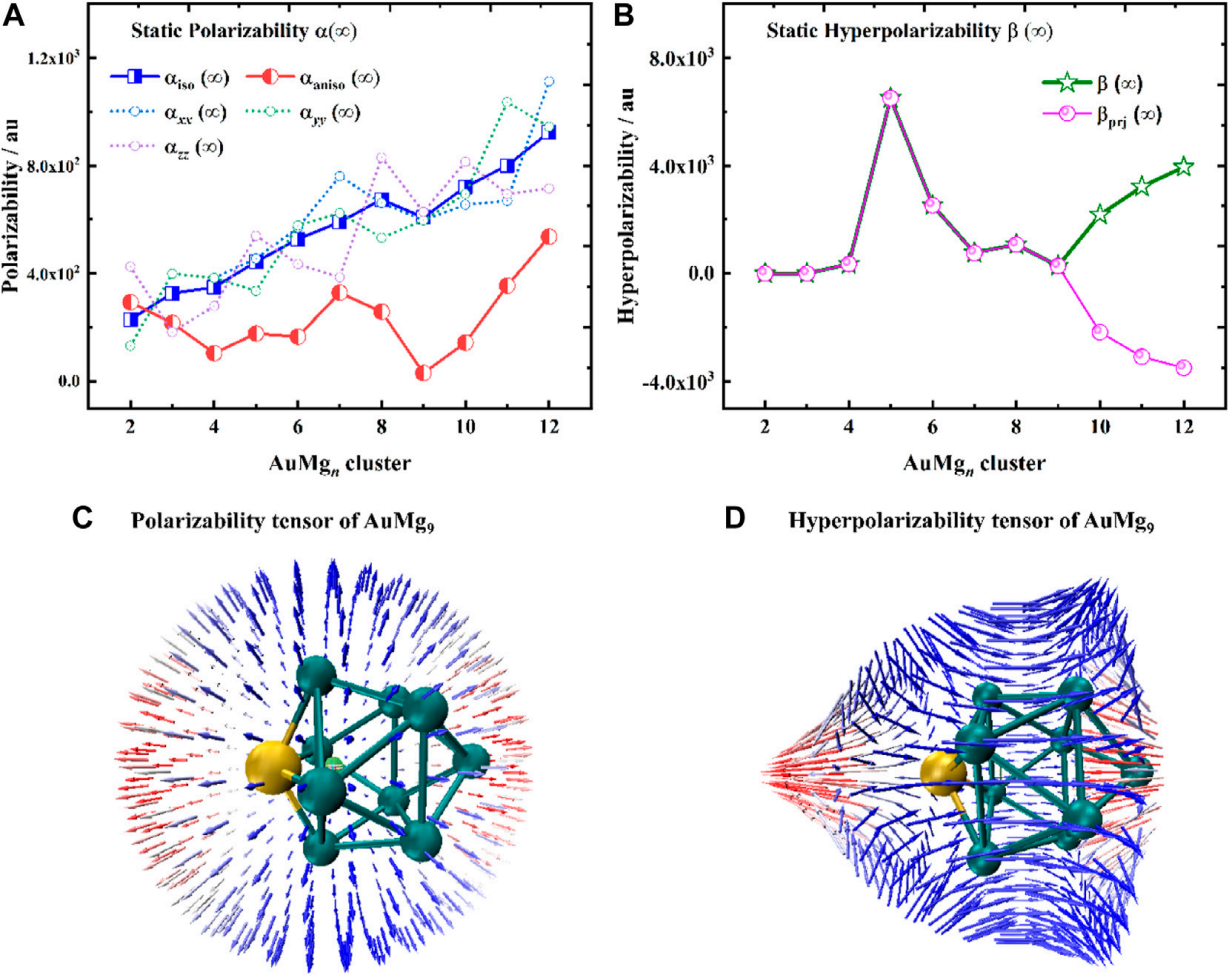
**(A)** Static polarizabilities α(∞), **(B)** Static hyperpolarizabilities β(∞) for the ground state of AuMg_n_ (*n* = 2–12) nanoclusters, **(C)** The unit sphere representation of static polarizability tensor α, **(D)** The unit sphere representation of static hyperpolarizability tensor β of AuMg_9_ nanocluster.

Static polarizabilities α (∞) and hyperpolarizabilities β (∞) for the ground state AuMg_n_ (*n* = 2–20) nanoclusters were calculated to analyze their nonlinear optical property. Specifically, the coupled-perturbed Kohn-Sham (CPKS) method (Jensen, 2017) was adopted for the AuMg_n_ nanoclusters to compute the polarizabilities and hyperpolarizabilities in the zero-frequency limit (λ→∞). The results of α(∞) and β(∞) calculations are presented in Supplementary Table S3 in the Supplementary Material and are shown in Figure 6. From Figure 6A, it can be seen that the polarization anisotropy α_aniso_ (∞) and isotropy α_iso_ (∞) of AuMg_n_ nanoclusters do not change consistently with the size. The α_iso_ (∞) shows an overall upward trend, except for AuMg_9_, while the α_aniso_ (∞) oscillates with increasing size. In addition, α_xx_ (∞), α_yy_ (∞), α_zz_ (∞) of each nanocluster are also shown in Figure 6A. Due to the diversity of the nanoclusters structures, these quantities display irregular oscillations in different directions. However, AuMg_9_ nanocluster with high structural symmetry exhibits synchronous local minimum anisotropic and isotropic polarization, suggesting that it has special nonlinear optical properties compared to other nanoclusters. In order to study the polarization of AuMg_9_ more intuitively, the unit sphere representation of its polarization tensor is plotted in Figure 6C. One can find the anisotropic polarization of AuMg_9_, more specifically, the small polarization rate in the x-y plane and the maximum polarization rate in the z-direction (i.e., the direction of the line connecting the leftmost Au and the rightmost Mg in the figure).

Figure 6B exhibits the static hyperpolarizability β (∞) of the AuMg_n_ nanoclusters and their projection values in the dipole moment direction β_prj_ (∞). Since β_prj_ (∞) can be measured by the electric field-induced second harmonic generation experiment (EFISH), it serves as a guide for experiments. Specifically, β increases from AuMg_2_ to a maximum value of AuMg_5_, then gradually decreases to a minimum value of AuMg_9_, and increases again afterward. Interestingly, the β and β_prj_ of AuMg_9_ and the nanoclusters smaller than it are exactly equal, indicating that its β is isotropic with the dipole moment. However, starting from AuMg_10_ nanocluster, the two curves are reversed, forming a mirror-symmetric trend. Figure 6D shows the unit sphere representation of static hyperpolarizability tensor β of AuMg_9_, it is found that β is also anisotropic, with a maximum in the z-direction, and changes in the x-y plane as the Mg atoms surround it. In conclusion, relative to other nanoclusters, AuMg9 exhibits distinctive nonlinear optical properties.

### Boltzmann Distribution Weighted Average Spectra of IR and Raman

**FIGURE 7:**
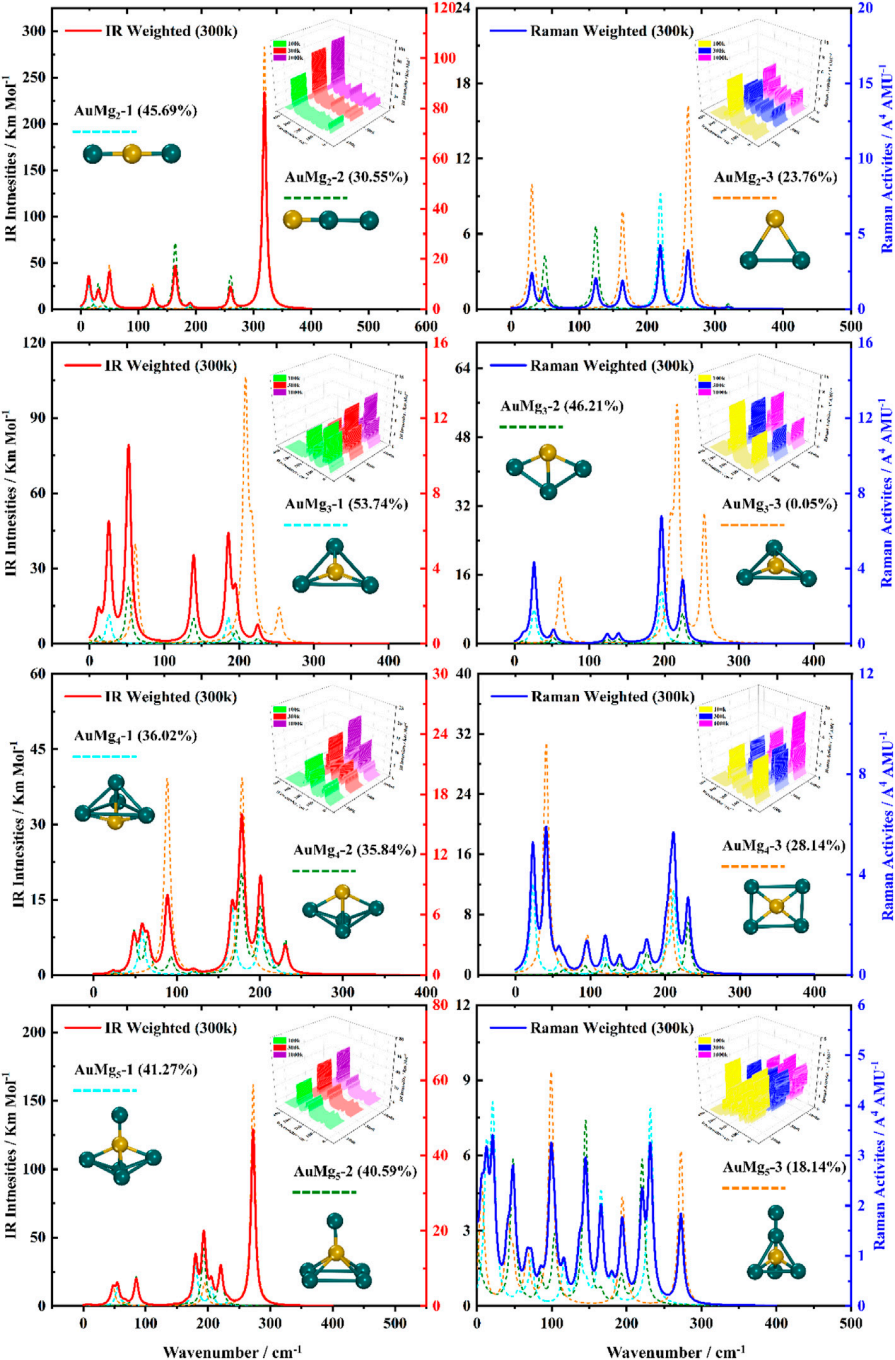
Boltzmann distribution weighted average spectra of the three lowest energy isomers of AuMg_n_ (*n* = 2–5) nanoclusters at room temperature (IR on the left side, Raman on the right side).

**FIGURE 8:**
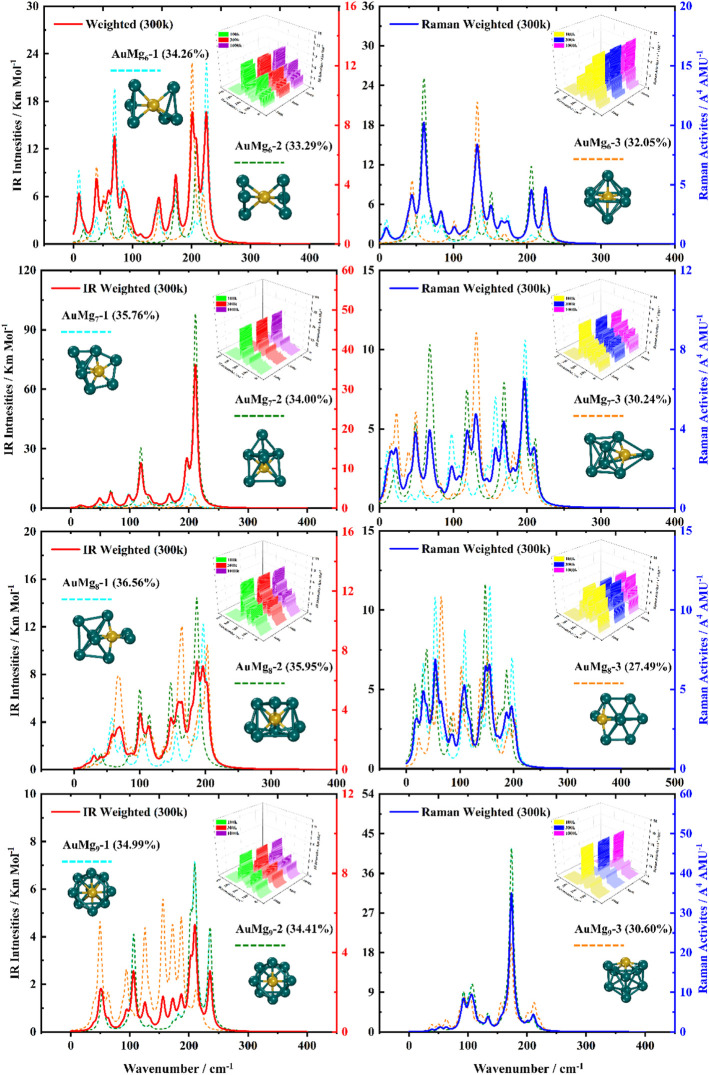
Boltzmann distribution weighted average spectra of the three lowest energy isomers of AuMg_n_ (*n* = 6–9) nanoclusters at room temperature (IR on the left side, Raman on the right side).

**FIGURE 9:**
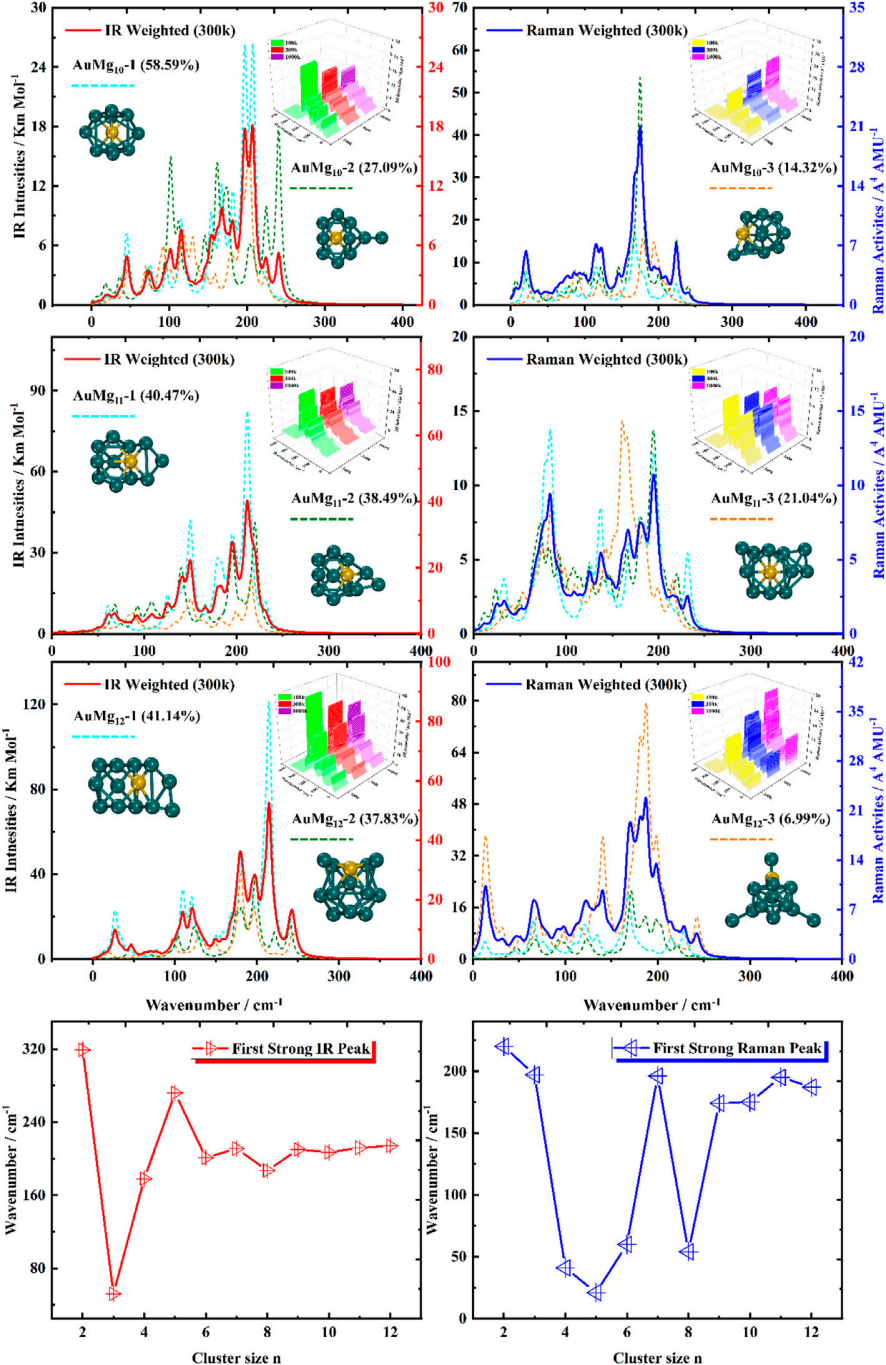
Boltzmann distribution weighted average spectra of the three lowest energy isomers of AuMg_n_ (*n* = 10–12) nanoclusters at room temperature (IR on the left side, Raman on the right side), and the first strong IR and Raman peaks in AuMg_n_ (*n* = 2–12) nanoclusters.

For the ground state AuMg_n_ nanoclusters, the infrared and Raman spectra with weighted average of the Boltzmann distribution were calculated for guidance experiments. The motivation for considering the Boltzmann distribution is due to the difficulty of observing only the ground state nanoclusters in experiments, especially in the gas-phase nanoclusters. Figure 7 display the IR and Raman spectra of the weighted average of the Boltzmann distribution at room temperature. The Boltzmann distribution probabilities of each isomer at different temperatures were also calculated by the relevant equations in the Supplementary Material. The small 3D plots in each map are the corresponding weighted average spectra at 100 k, 300 k and 1000 k temperatures. As shown in Figures 7–9, the strongest absorption peaks of IR spectra are distributed in the 40–350 cm^−1^ frequency band, while the strongest peaks of Raman spectra are distributed in a narrower band of 20–220 cm^−1^. However, the most intense peaks of both IR and Raman weighted average spectra appear around 200 cm^−1^ as the size increases. In addition, as can be seen from the small 3D plots in each figure and Supplementary Figure S2 in the Supplementary Material, the location of the most intense peak of the weighted average spectrum does not shift as the temperature increases, but the intensity changes.

Specifically, for the weighted average IR spectra at room temperature, the nanoclusters of AuMg_n_ (*n* = 4–12) are easily distinguished as separate strong bands in the 200 cm^−1^ regions of the spectra, except for the AuMg_2_ and AuMg_3_ nanoclusters. This result is in good agreement with the results of infrared spectroscopy of pure Mg nanoclusters studied by Belyaev et al. (Belyaev et al., 2016). For the Raman weighted average spectrum at room temperature, although the strongest Raman activity peaks of AuMg_4_, AuMg_5_ and AuMg_6_ nanoclusters appear in the low-frequency band (20–50 cm^−1^), they still have many strong peaks in the 200 cm^−1^ regions. Therefore, for all the Raman spectra of the AuMg_n_ (*n* = 2–12) nanoclusters, the 200 cm^−1^ regions can be more easily distinguished as individual strong bands. In conclusion, it was computationally shown that the formation of AuMg_n_ (*n* = 2–12) nanoclusters at room temperature is possible to identify these nanoclusters by IR and Raman spectroscopy.

### The Average Bond Distance and Average Nearest Neighbor Distance

**FIGURE 10:**
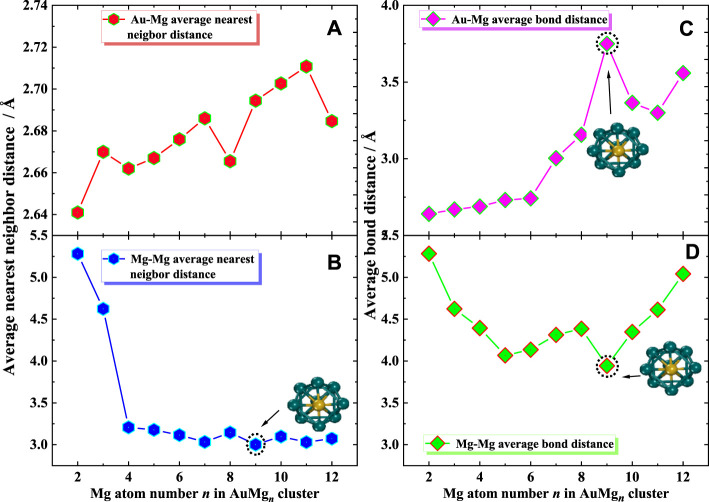
**(A,B)** Average nearest neighbor distance for Au-Mg and Mg-Mg, **(C,D)** Average bond distance for Au-Mg and Mg-Mg.

In order to provide more data support for future possible experiments, the average bond distance and average nearest neighbor distance were calculated. As shown in Figure 10, the average nearest neighbor distance and bond distance for Au-Mg and Mg-Mg in the ground state of AuMg_n_ (*n* = 2–12) clusters display some interesting conclusions. Figures 10A,B show that, overall, the nearest neighbor distance for Au-Mg becomes larger as the cluster size increases (except for *n* = 8 and 12), while Mg-Mg is overall decreasing. The average nearest neighbor distance of Au-Mg is 2.68 Å, and that of Mg-Mg is 3.44 Å. Figure 10C gives the average bond distance of Au-Mg with cluster size dependence similar to the nearest neighbor distance of Au-Mg, i.e., increasing overall. However, Figure 10D demonstrates that the average Mg-Mg bond distance decreases and then increases with cluster size. Another interesting conclusion is that the local turning points of the average bond and nearest-neighbor distances for Mg-Mg and the average bond distance curves for Au-Mg occur at AuMg_9_, suggesting that they influence the local stability of the clusters.

## Conclusion

In this work, the structure of Au-doped Mg_n_ (*n* = 2–12) nanoclusters was investigated by CALYPSO crystal search software. It is shown that the geometric growth mechanism of this nanocluster has similarities to other atom-doped Mg_n_ clusters but also has unique features, such as the planar structure of AuMg_3_ and the high symmetry cage-like structure of AuMg_9_. Stability calculations show that AuMg_4_ and AuMg_10_ have high local stability, while AuMg_9_ nanoclusters are the second most stable nanoclusters. The charge transfer study reveals that Au atoms are electron receivers and Mg atoms are electron donors in AuMg_n_ nanoclusters. ELF analysis showed that Mg-Mg formed a covalent chemical bond while Au-Mg was an ionic bond, and the critical size for the appearance of the Mg-Mg covalent bond was found to be AuMg_3_. The nonlinear optical properties of AuMg_n_ nanoclusters were probed by calculating the static polarizability and hyperpolarizability, and the results indicate that AuMg_9_ is a special one of interest. Boltzmann distribution weighted average IR and Raman spectroscopy studies at room temperature confirm that these nanoclusters can be identified by spectroscopic experiments. Finally, the average bond distance and average nearest neighbor distance were fully investigated.

## Data Availability

The original contributions presented in the study are included in the article/Supplementary Material, further inquiries can be directed to the corresponding authors.

## References

[B1] Alvarez M. M. Khoury J. T. Schaaff T. G. Shafigullin M. Vezmar I. Whetten R. L. (1997). Critical Sizes in the Growth of Au Clusters. Chem. Phys. Lett. 266, 91–98. 10.1016/S0009-2614(96)01535-7

[B2] Assadollahzadeh B. Schwerdtfeger P. (2009). A Systematic Search for Minimum Structures of Small Gold Clusters Au[sub N] (N=2-20) and Their Electronic Properties. J. Chem. Phys. 131, 064306. 10.1063/1.3204488 19691387

[B3] Becke A. D. Edgecombe K. E. (1990). A Simple Measure of Electron Localization in Atomic and Molecular Systems. J. Chem. Phys. 92, 5397–5403. 10.1063/1.458517

[B4] Belyaev S. N. Panteleev S. V. Ignatov S. K. Razuvaev A. G. (2016). Structural, Electronic, Thermodynamic and Spectral Properties of Mgn (N=2-31) Clusters. A DFT Study. Comput. Theor. Chem. 1079, 34–46. 10.1016/j.comptc.2016.01.011

[B5] Chen B. Conway L. J. Sun W. Kuang X. Lu C. Hermann A. (2021). Phase Stability and Superconductivity of lead Hydrides at High Pressure. Phys. Rev. B 103, 035131. 10.1103/PhysRevB.103.035131

[B6] Chen H. Liang H. Dai W. Lu C. Ding K. Bi J. (2020). MgScH15: A Highly Stable Cluster for Hydrogen Storage. Int. J. Hydrogen Energ. 45, 32260–32268. 10.1016/j.ijhydene.2020.08.229

[B7] Frisch M. J. Trucks G. W. Schlegel H. B. Scuseria G. E. Robb M. A. Cheeseman J. R. (2016). Gaussian 09, Revision A.02. Wallingford, CT: Gaussian, Inc. Available at: https://gaussian.com/g09citation/ .

[B8] Huang W. Wang L.-S. (2009). Probing the 2D to 3D Structural Transition in Gold Cluster Anions Using Argon Tagging. Phys. Rev. Lett. 102, 153401. 10.1103/PhysRevLett.102.153401 19518630

[B9] Idrobo J. C. Walkosz W. Yip S. F. Öğüt S. Wang J. Jellinek J. (2007). Static Polarizabilities and Optical Absorption Spectra of Gold Clusters (Aun,n=2-14and 20) from First Principles. Phys. Rev. B 76, 205422. 10.1103/PhysRevB.76.205422

[B10] Jellinek J. Acioli P. H. (2003). Magnesium Clusters: Structural and Electronic Properties and the Size-Induced Nonmetal-To-Metal Transition. J. Phys. Chem. A. 107, 1670. 10.1021/jp0301655

[B11] Jensen F. (2017). Introduction to Computational Chemistry. West Sussex: John Wiley & Sons. Available at: https://xs.dailyheadlines.cc/books/about/Introduction_to_Computational_Chemistry.html?hl=zh-CN&id=UZOVDQAAQBAJ (Accessed October 14, 2021).

[B12] Jin R. (2015). Atomically Precise Metal Nanoclusters: Stable Sizes and Optical Properties. Nanoscale 7, 1549–1565. 10.1039/C4NR05794E 25532730

[B13] Jin R. Zeng C. Zhou M. Chen Y. (2016). Atomically Precise Colloidal Metal Nanoclusters and Nanoparticles: Fundamentals and Opportunities. Chem. Rev. 116, 10346–10413. 10.1021/acs.chemrev.5b00703 27585252

[B14] Köhn A. Weigend F. Ahlrichs R. (2001). Theoretical Study on Clusters of Magnesium. Phys. Chem. Chem. Phys. 3, 711–719. 10.1039/B007869G

[B15] Li Z. Zhao Z. Zhou Z. Wang H. Li S. (2017). First-principles Calculations on Small MgnZn and Mgn-1Zn2 Clusters: Structures, Stability, Electronic Properties. Mater. Chem. Phys. 199, 585–590. 10.1016/j.matchemphys.2017.07.049

[B16] Lu C. Chen C. (2021). Indentation Strengths of Zirconium Diboride: Intrinsic versus Extrinsic Mechanisms. J. Phys. Chem. Lett. 12, 2848–2853. 10.1021/acs.jpclett.1c00434 33720728

[B17] Lu C. Chen C. (2020a). Indentation-strain Stiffening in Tungsten Nitrides: Mechanisms and Implications. Phys. Rev. Mater. 4, 043402. 10.1103/PhysRevMaterials.4.043402

[B18] Lu C. Chen C. (2020b). Structure-strength Relations of Distinct Mon Phases from First-Principles Calculations. Phys. Rev. Mater. 4, 044002. 10.1103/PhysRevMaterials.4.044002

[B19] Lu C. Gong W. Li Q. Chen C. (2020). Elucidating Stress-Strain Relations of ZrB12 from First-Principles Studies. J. Phys. Chem. Lett. 11, 9165–9170. 10.1021/acs.jpclett.0c02656 33054239

[B20] Lu T. Chen F. (2012). Multiwfn: A Multifunctional Wavefunction Analyzer. J. Comput. Chem. 33, 580–592. 10.1002/jcc.22885 22162017

[B21] Lv J. Wang Y. Zhu L. Ma Y. (2012). Particle-swarm Structure Prediction on Clusters. J. Chem. Phys. 137, 084104. 10.1063/1.4746757 22938215

[B22] Lyon J. T. (2021). Hydrogen Binding and Dissociation in MgScH Clusters (N ≤ 20). Int. J. Hydrogen Energ. 46, 36872–36877. 10.1016/j.ijhydene.2021.08.228

[B23] Peng Y. Wang P. Luo L. Liu L. Wang F. (2018). Green Synthesis of Fluorescent Palladium Nanoclusters. Materials 11, 191. 10.3390/ma11020191 PMC5848888 29373486

[B24] Qian H. Jin R. (2009). Controlling Nanoparticles with Atomic Precision: The Case of Au144(SCH2CH2Ph)60. Nano Lett. 9, 4083–4087. 10.1021/nl902300y 19995083

[B25] Ramakrishna G. Varnavski O. Kim J. Lee D. Goodson T. (2008). Quantum-Sized Gold Clusters as Efficient Two-Photon Absorbers. J. Am. Chem. Soc. 130, 5032–5033. 10.1021/ja800341v 18357982

[B26] Reed A. E. Curtiss L. A. Weinhold F. (1988). Intermolecular Interactions from a Natural Bond Orbital, Donor-Acceptor Viewpoint. Chem. Rev. 88, 899–926. 10.1021/cr00088a005

[B27] Shang L. Dong S. Nienhaus G. U. (2011). Ultra-small Fluorescent Metal Nanoclusters: Synthesis and Biological Applications. Nano Today 6, 401–418. 10.1016/j.nantod.2011.06.004

[B28] Shao H. Xin G. Zheng J. Li X. Akiba E. (2012). Nanotechnology in Mg-Based Materials for Hydrogen Storage. Nano Energy 1, 590–601. 10.1016/j.nanoen.2012.05.005

[B29] Shinde R. (2016). Ab Initio Calculations of Optical Properties of Clusters. ArXiv160706928 Phys Available at: http://arxiv.org/abs/1607.06928 (Accessed October 11, 2021).

[B30] Shinde R. Shukla A. (2017). First Principles Electron-Correlated Calculations of Optical Absorption in Magnesium Clusters. Eur. Phys. J. D 71, 301. 10.1140/epjd/e2017-80356-6

[B31] Sun W. Kuang X. Keen H. D. J. Lu C. Hermann A. (2020). Second Group of High-Pressure High-Temperature Lanthanide Polyhydride Superconductors. Phys. Rev. B 102, 144524. 10.1103/PhysRevB.102.144524

[B32] Tew L. Cai M.-T. Lo L.-W. Khung Y. Chen N.-T. (2018). Pollen-Structured Gold Nanoclusters for X-ray Induced Photodynamic Therapy. Materials 11, 1170. 10.3390/ma11071170 PMC6073926 29987236

[B33] Trivedi R. Bandyopadhyay D. (2015). Hydrogen Storage in Small Size MgnCo Clusters: A Density Functional Study. Int. J. Hydrogen Energ. 40, 12727–12735. 10.1016/j.ijhydene.2015.07.122

[B34] Trivedi R. Bandyopadhyay D. (2016). Study of Adsorption and Dissociation Pathway of H 2 Molecule on Mg N Rh (N = 1-10) Clusters: A First Principle Investigation. Int. J. Hydrogen Energ. 41, 20113–20121. 10.1016/j.ijhydene.2016.09.007

[B35] Wang Y. Lv J. Zhu L. Ma Y. (2012). CALYPSO: A Method for crystal Structure Prediction. Comput. Phys. Commun. 183, 2063–2070. 10.1016/j.cpc.2012.05.008

[B36] Wang Y. Lv J. Zhu L. Ma Y. (2010). Crystal Structure Prediction via Particle-Swarm Optimization. Phys. Rev. B 82, 094116. 10.1103/PhysRevB.82.094116

[B37] Xia X. Kuang X. Lu C. Jin Y. Xing X. Merino G. (2016). Deciphering the Structural Evolution and Electronic Properties of Magnesium Clusters: An Aromatic Homonuclear Metal Mg17 Cluster. J. Phys. Chem. A. 120, 7947–7954. 10.1021/acs.jpca.6b07322 27607143

[B38] Yan N. Xia N. Liao L. Zhu M. Jin F. Jin R. (2018). Unraveling the Long-Pursued Au 144 Structure by X-ray Crystallography. Sci. Adv. 4, eaat7259. 10.1126/sciadv.aat7259 30333988 PMC6184749

[B39] Yau S. H. Varnavski O. Goodson T. (2013). An Ultrafast Look at Au Nanoclusters. Acc. Chem. Res. 46, 1506–1516. 10.1021/ar300280w 23651457

[B40] Zeng L. Liang M.-K. Wei X.-F. Guo J. Dai W. Zhu B.-C. (2021). New Potential Stable Structures of XMg N (X = Ge, C, Sn; N = 2-12) Clusters: XMg8 with High Stability. J. Phys. Condens. Matter 33, 065302. 10.1088/1361-648X/abc401 33091897

[B41] Zeng L. Wei X.-F. Liang M.-K. Deng P.-J. Bi J. Zhu B.-C. (2020). BeMg9: A tower-like Type Doped Magnesium Clusters with High Stability. Comput. Mater. Sci. 182, 109795. 10.1016/j.commatsci.2020.109795

[B42] Zhang F. Zhang H. Xin W. Chen P. Hu Y. Zhang X. (2020). Probing the Structural Evolution and Electronic Properties of Divalent Metal Be2Mgn Clusters from Small to Medium-Size. Sci. Rep. 10, 6052. 10.1038/s41598-020-63237-8 32269297 PMC7142069

[B43] Zhao Y. R. Bai T. T. Jia L. N. Xin W. Hu Y. F. Zheng X. S. (2019). Probing the Structural and Electronic Properties of Neutral and Anionic Lanthanum-Doped Silicon Clusters. J. Phys. Chem. C 123, 28561–28568. 10.1021/acs.jpcc.9b07184

[B44] Zhao Y. Xu Y. Chen P. Yuan Y. Qian Y. Li Q. (2021). Structural and Electronic Properties of Medium-Sized Beryllium Doped Magnesium BeMg Clusters and Their Anions. Results Phys. 26, 104341. 10.1016/j.rinp.2021.104341

[B45] Zhu B.-C. Deng P.-J. Xiong S.-Y. Dai W. Zeng L. Guo J. (2021). Au5Br: A New Member of Highly Stable 2D-type Doped Gold Nanomaterial. Comput. Mater. Sci. 194, 110446. 10.1016/j.commatsci.2021.110446

[B46] Zhu B. C. Zhang S. Zeng L. (2020). The Effect of Silicon Doping on the Geometrical Structures, Stability, and Electronic and Spectral Properties of Magnesium Clusters: DFT Study of SiMg N ( N = 1‐12) Clusters. Int. J. Quan. Chem. 120, e26143. 10.1002/qua.26143

