# Supplementary material for: Computational Exploration on the Structural and Optical Properties of Gold-Doped Alkaline-Earth Magnesium AuMgn (n = 2–12) Nanoclusters: DFT Study
Source: Front Chem. 2022 Mar 29;10:870985. doi: 10.3389/fchem.2022.870985 (PMC9001918; doi:10.3389/fchem.2022.870985)
Supplement: Supplementary file 1 [file DataSheet1.docx]

**Supplementary Materials**

**Computational exploration on structure and optical properties of gold-doped** **alkaline-earth magnesium AuMg_n_ (n=2-12) nanoclusters: DFT study**

Ben-Chao Zhu*^1^, Ping-Ji Deng^1^, Jia Guo^1^, Wen-Bin Kang*^1^

^1^School of Public Health, Hubei University of Medicine, Shiyan 442000, China

| **Table of Contents** | **Pages** |
| --- | --- |
| **Table S1.** Opted atomic coordinate in the ground state of AuMg_n_ (n=2-12) nanoclusters. | 2–12 |
| **Table S2.** NCP on Mg atoms in the ground state of AuMgn (n=2-12) nanoclusters. | 4–12 |
| **Table S3.** Polarizabilities and first-order hyperpolarizabilities in the ground state of AuMg_n_ (n=2-12) nanoclusters. | 5–12 |
| **Figure S1.** The ELF analysis for Mg-Mg and Au-Mg chemical bonds for AuMgn (n=2, 5-8, 11, 12) nanoclusters. | 6–12 |
| **Figure S2.** Weighted average spectra of the Boltzmann distribution of the three lowest energy isomers of AuMg_n_ (n=2-12) nanoclusters at 100k and 1000k temperatures (IR on the left, Raman on the right). | 7–12 |
|  |  |

**Table S1**. Opted atomic coordinate in the ground state of AuMg_n_ (n=2-12) nanoclusters.

| AuMg_2_ | X | Y | Z | AuMg_8_ | X | Y | Z |
| --- | --- | --- | --- | --- | --- | --- | --- |
| Au-1 | 0.00000000 | 0.00000000 | 0.00000000 | Au-1 | 0.00000000 | 0.00000000 | 0.67984700 |
| Mg-2 | 0.00000000 | 0.00000000 | 2.64094900 | Mg-2 | 0.00000000 | 1.58746200 | -1.58570000 |
| Mg-3 | 0.00000000 | 0.00000000 | -2.64094900 | Mg-3 | 0.00000000 | -1.88355300 | 2.56577800 |
| AuMg_3_ | X | Y | Z | Mg-4 | 2.63823500 | 0.00000000 | 0.03473300 |
| Au-1 | 0.00000000 | 0.00000000 | 0.00000000 | Mg-5 | -2.63823500 | 0.00000000 | 0.03473300 |
| Mg-2 | 0.00000000 | 2.66981900 | 0.00000000 | Mg-6 | 0.00000000 | -1.58746200 | -1.58570000 |
| Mg-3 | 2.31213100 | -1.33490900 | 0.00000000 | Mg-7 | 0.00000000 | 1.88355300 | 2.56577800 |
| Mg-4 | -2.31213100 | -1.33490900 | 0.00000000 | Mg-8 | -2.14382100 | 0.00000000 | -3.25264000 |
| AuMg_4_ | X | Y | Z | Mg-9 | 2.14382100 | 0.00000000 | -3.25264000 |
| Au-1 | 0.00000000 | 0.00000000 | 0.49018700 | AuMg_9_ | X | Y | Z |
| Mg-2 | 0.00000000 | 2.53665600 | -0.31616500 | Au-1 | 0.000000 | 0.000000 | 1.615040 |
| Mg-3 | 2.19680900 | -1.26832800 | -0.31616500 | Mg-2 | 1.631442 | -1.631442 | -2.061844 |
| Mg-4 | -2.19680900 | -1.26832800 | -0.31616500 | Mg-3 | 0.000000 | 2.404641 | 0.399349 |
| Mg-5 | 0.00000000 | 0.00000000 | -2.27857200 | Mg-4 | 2.404641 | 0.000000 | 0.399349 |
| AuMg_5_ | X | Y | Z | Mg-5 | 1.631442 | 1.631442 | -2.061844 |
| Au-1 | 0.00000000 | 0.00000000 | 0.31431600 | Mg-6 | 0.000000 | -2.404641 | 0.399349 |
| Mg-2 | 0.00000000 | 0.00000000 | 3.13623700 | Mg-7 | -1.631442 | 1.631442 | -2.061844 |
| Mg-3 | 0.00000000 | 1.67704000 | -1.86497500 | Mg-8 | -2.404641 | 0.000000 | 0.399349 |
| Mg-4 | 0.00000000 | -1.67704000 | -1.86497500 | Mg-9 | 0.000000 | 0.000000 | -3.982367 |
| Mg-5 | 2.45107200 | 0.00000000 | -0.73776800 | Mg-10 | -1.631442 | -1.631442 | -2.061844 |
| Mg-6 | -2.45107200 | 0.00000000 | -0.73776800 | AuMg_10_ | X | Y | Z |
| AuMg_6_ | X | Y | Z | Au-1 | 0.000000 | 0.000000 | 1.018093 |
| Au-1 | 0.00000000 | 0.00000000 | 0.15711100 | Mg-2 | -1.644406 | 1.768245 | -0.232597 |
| Mg-2 | 0.00000000 | 2.79589800 | 0.26402500 | Mg-3 | 0.000000 | 1.614869 | -2.875144 |
| Mg-3 | 1.55379600 | 0.84790500 | -1.85000600 | Mg-4 | -1.644406 | -1.768245 | -0.232597 |
| Mg-4 | -2.55917100 | -0.43386200 | 1.06882300 | Mg-5 | 0.000000 | -2.234727 | 2.538108 |
| Mg-5 | 2.55917100 | 0.43386200 | 1.06882300 | Mg-6 | 2.692902 | 0.000000 | -2.548994 |
| Mg-6 | 0.00000000 | -2.79589800 | 0.26402500 | Mg-7 | 1.644406 | 1.768245 | -0.232597 |
| Mg-7 | -1.55379600 | -0.84790500 | -1.85000600 | Mg-8 | 1.644406 | -1.768245 | -0.232597 |
| AuMg_7_ | X | Y | Z | Mg-9 | 0.000000 | 2.234727 | 2.538108 |
| Au-1 | -0.45205800 | 0.03260900 | 0.07067400 | Mg-10 | 0.000000 | -1.614869 | -2.875144 |
| Mg-2 | 1.37098600 | -1.38344800 | -1.47885400 | Mg-11 | -2.692902 | 0.000000 | -2.548994 |
| Mg-3 | 3.88997000 | 0.31791000 | -0.35238000 |  |  |  |  |
| Mg-4 | 1.45952600 | 2.12693000 | -0.22081100 |  |  |  |  |
| Mg-5 | -1.56281400 | 2.58741300 | 0.34631200 |  |  |  |  |
| Mg-6 | -2.98889000 | -0.28041500 | -0.75515500 |  |  |  |  |
| Mg-7 | -0.87848800 | -2.69024900 | 0.44814000 |  |  |  |  |
| Mg-8 | 1.68576100 | -0.89281600 | 1.54747800 |  |  |  |  |
|  |  |  |  |  |  |  |  |
|  |  |  |  |  |  |  |  |
| AuMg_11_ | X | Y | Z | AuMg_12_ | X | Y | Z |
| Au-1 | -0.356776 | 0.406785 | 0.000000 | Au-1 | 0.124224 | 0.385540 | 0.000000 |
| Mg-2 | 0.332423 | 2.339641 | 1.771339 | Mg-2 | -2.013853 | -2.292999 | 2.702891 |
| Mg-3 | -1.304941 | -0.361609 | -2.445359 | Mg-3 | -1.026933 | -3.556173 | 0.000000 |
| Mg-4 | -2.496592 | -1.890601 | 0.000000 | Mg-4 | -3.473407 | -1.568077 | 0.000000 |
| Mg-5 | 1.792032 | -0.444290 | -1.611492 | Mg-5 | 0.844099 | -1.667781 | 1.616505 |
| Mg-6 | -0.031315 | -3.003480 | 1.519198 | Mg-6 | 2.808449 | 0.335393 | 0.000000 |
| Mg-7 | 0.565485 | 4.994253 | 0.000000 | Mg-7 | 2.453305 | 3.743785 | 0.000000 |
| Mg-8 | 0.332423 | 2.339641 | -1.771339 | Mg-8 | 0.844099 | -1.667781 | -1.616505 |
| Mg-9 | -0.031315 | -3.003480 | -1.519198 | Mg-9 | -2.013853 | 0.624318 | 1.636227 |
| Mg-10 | 1.792032 | -0.444290 | 1.611492 | Mg-10 | 5.313944 | 2.187905 | 0.000000 |
| Mg-11 | -1.304941 | -0.361609 | 2.445359 | Mg-11 | -0.525952 | 2.991954 | 0.000000 |
| Mg-12 | 2.703486 | -2.842178 | 0.000000 | Mg-12 | -2.013853 | 0.624318 | -1.636227 |
|  |  |  |  | Mg-13 | -2.013853 | -2.292999 | -2.702891 |

**Table S2**. NCP on Mg atoms in the ground state of AuMg_n_ (n=2-12) nanoclusters.

**Table S3**. Polarizabilities and first-order hyperpolarizabilities in the ground state of AuMg_n_ (n=2-12) nanoclusters.

Isotropy α_iso_

$\alpha_{\text{iso}}=(\alpha_{xx}+\alpha_{yy}+\alpha_{zz})/3$ (S1)

Polarizability anisotropy α_aniso_

$\alpha_{aniso}=\sqrt{[(\alpha_{xx}-\alpha_{yy})^{2}-(\alpha_{yy}-\alpha_{zz})^{2}-(\alpha_{xx}-\alpha_{zz})^{2}+6(\alpha_{xy}^{2}+\alpha_{yz}^{2}+\alpha_{xz}^{2})]/2}$ (S2)


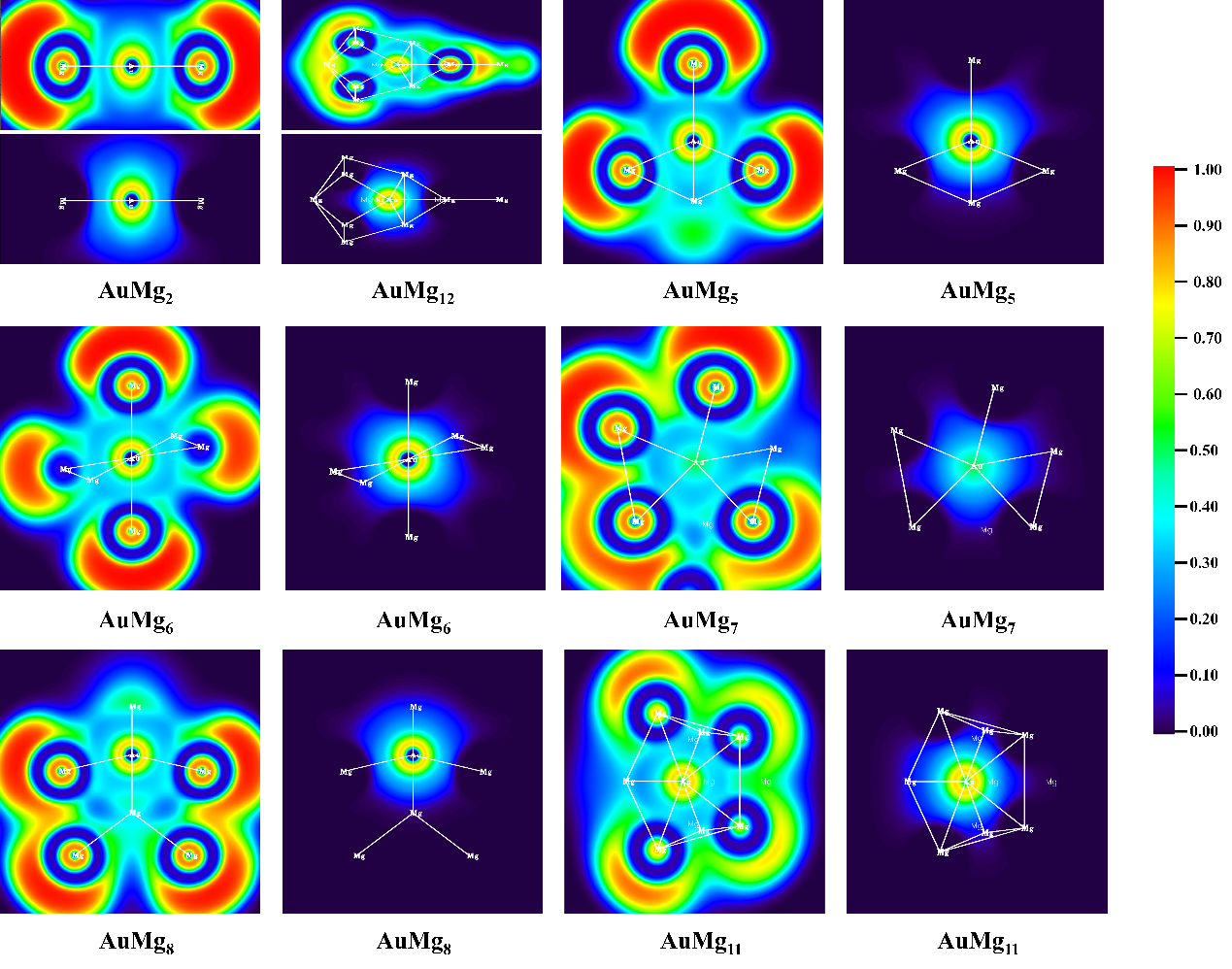


**Figure S1.** The ELF analysis for Mg-Mg and Au-Mg chemical bonds for AuMg_n_ (n=2, 5-8, 11, 12) nanoclusters.


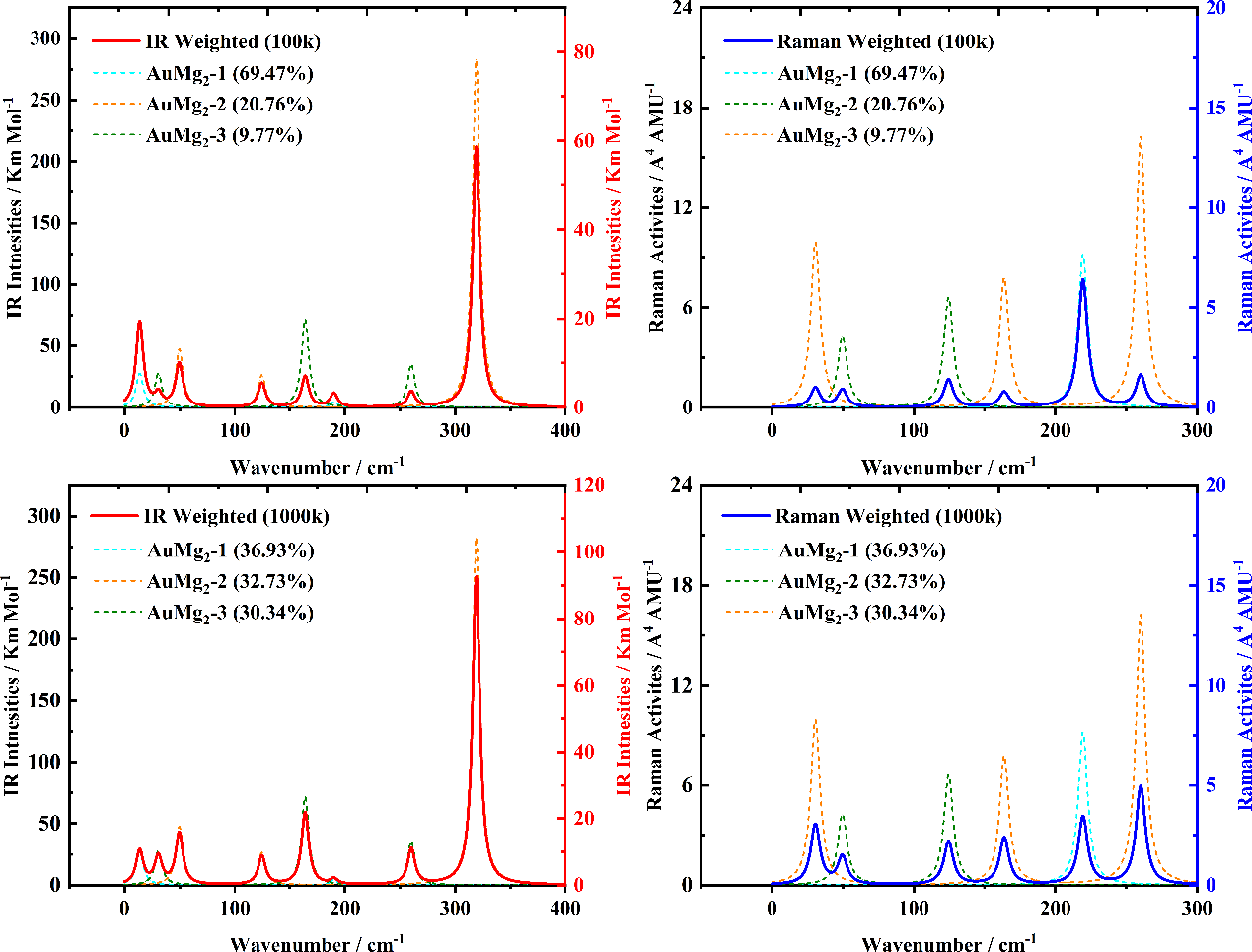


**Figure S2-1.** Weighted average spectra of AuMg_2_ nanocluster at 100k and 1000k temperatures.


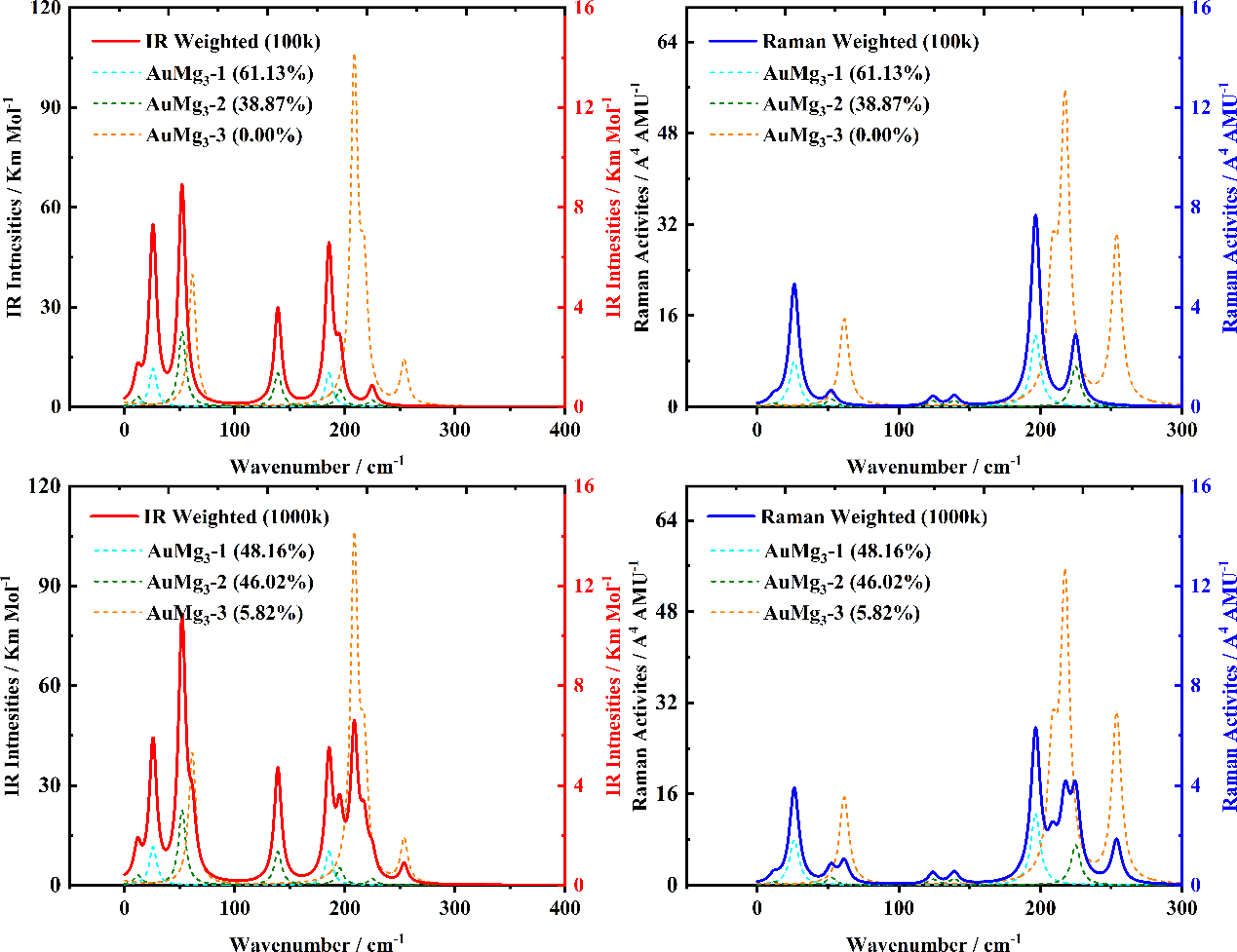


**Figure S2-2.** Weighted average spectra of AuMg_3_ nanocluster at 100k and 1000k temperatures.


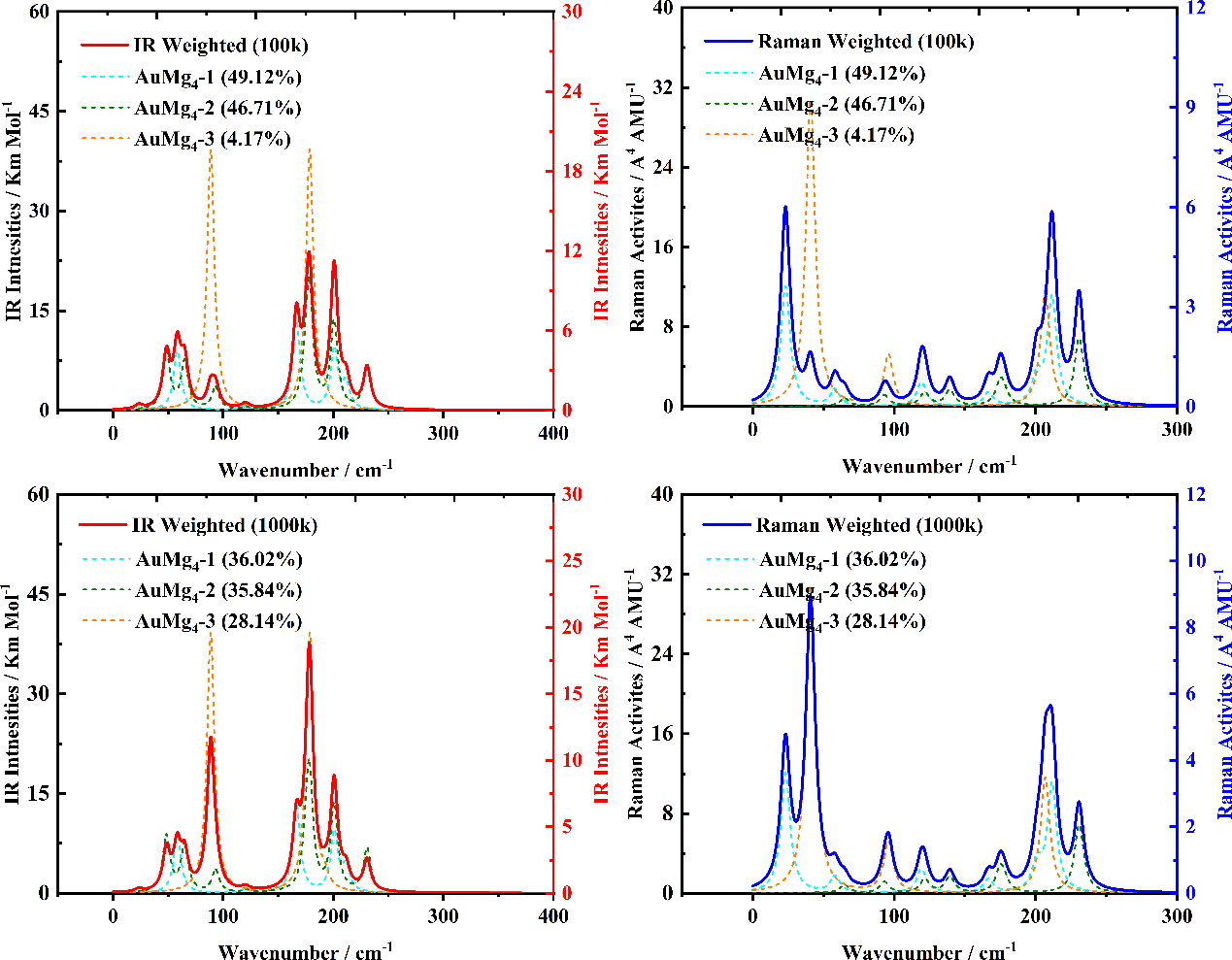


**Figure S2-3.** Weighted average spectra of AuMg_4_ nanocluster at 100k and 1000k temperatures.


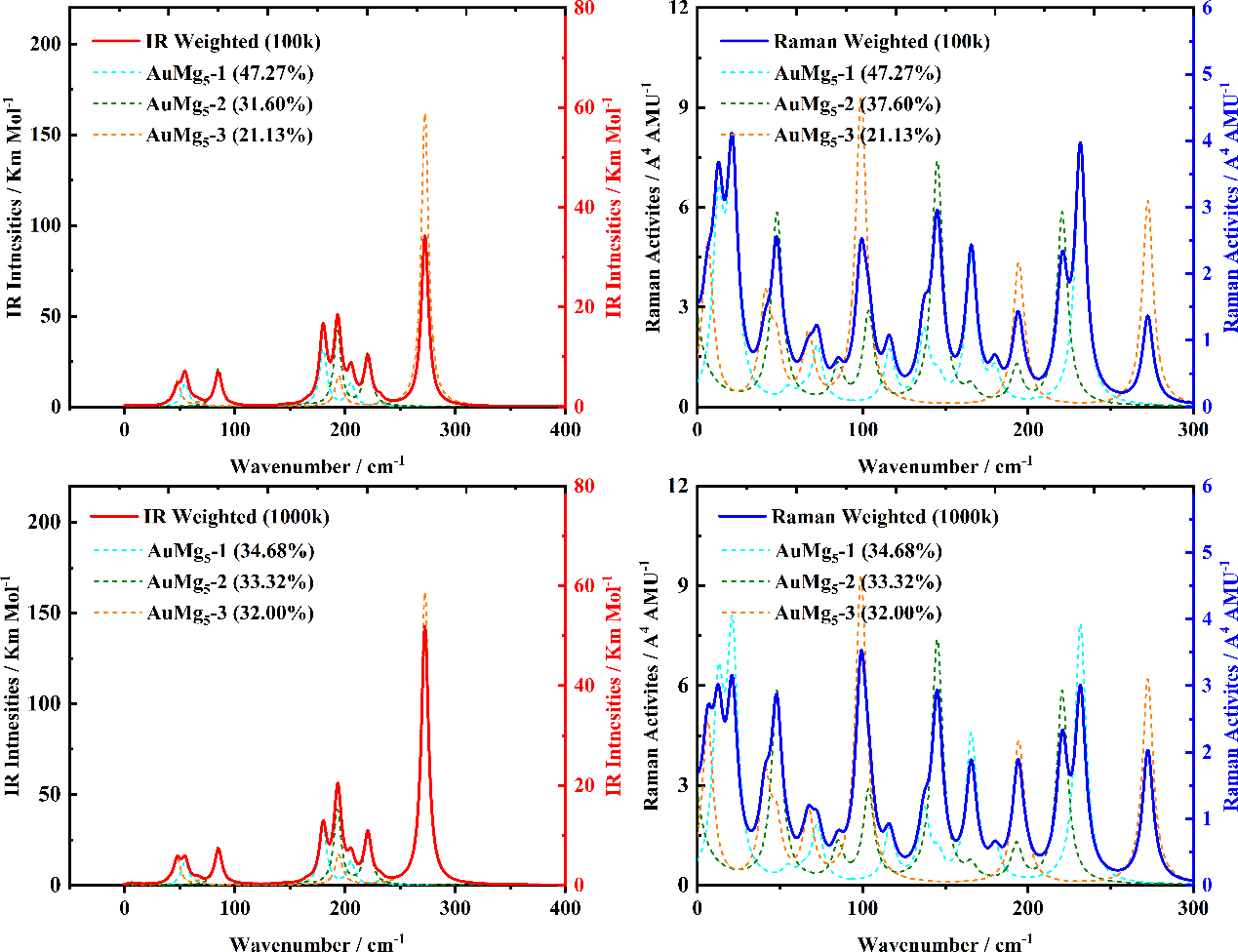


**Figure S2-4.** Weighted average spectra of AuMg_5_ nanocluster at 100k and 1000k temperatures.


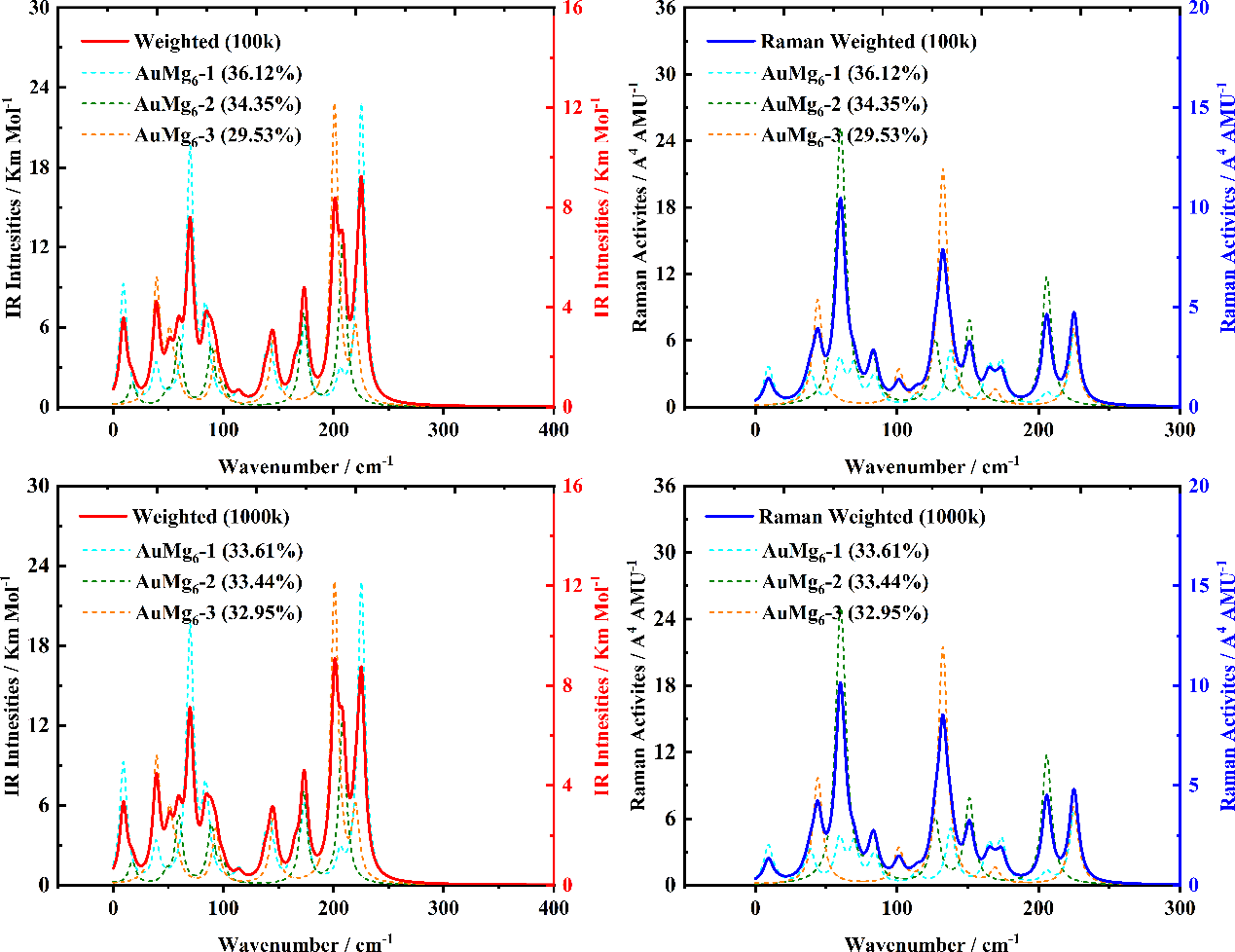


**Figure S2-5.** Weighted average spectra of AuMg_6_ nanocluster at 100k and 1000k temperatures.


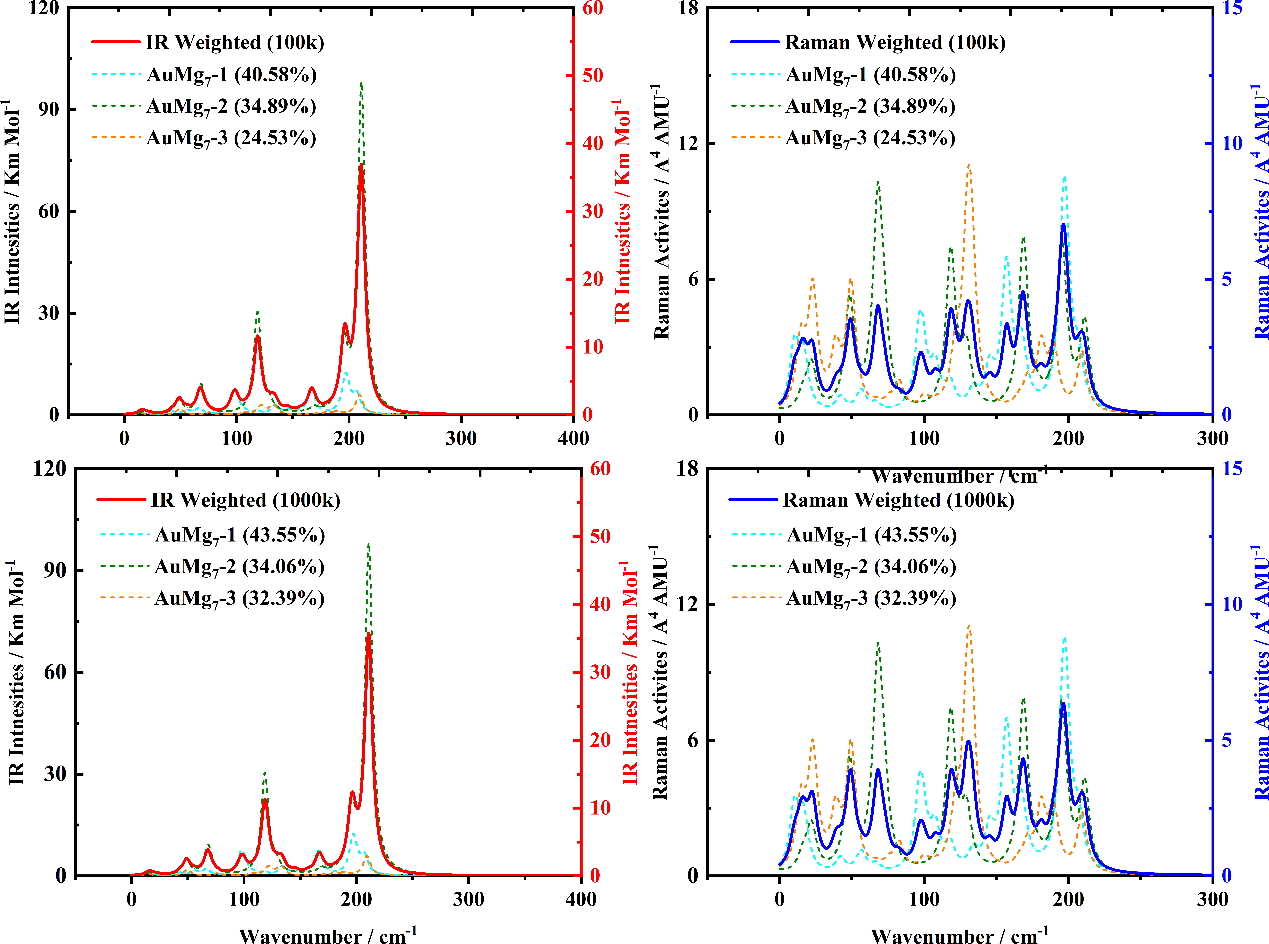


**Figure S2-6.** Weighted average spectra of AuMg_7_ nanocluster at 100k and 1000k temperatures.
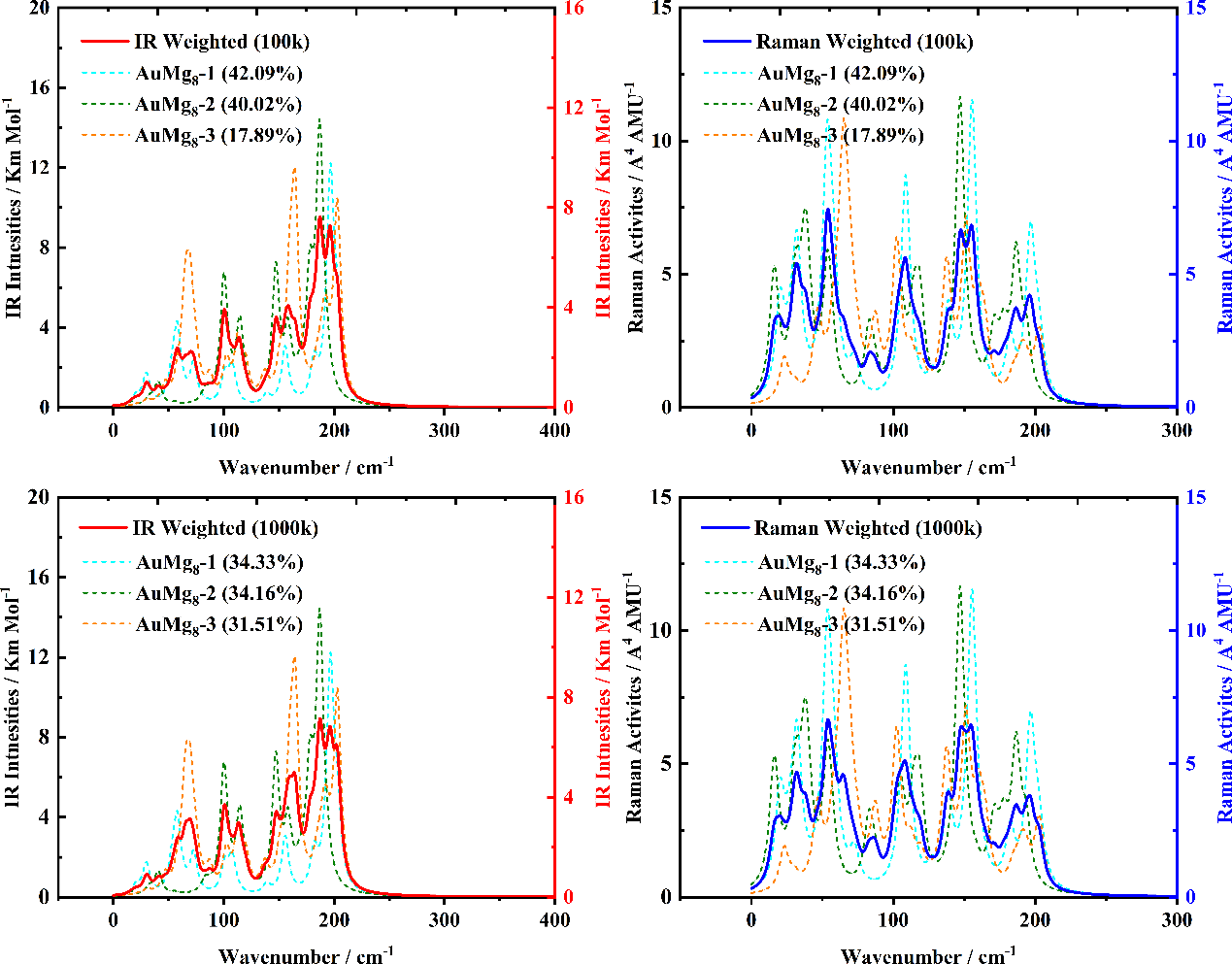


**Figure S2-7.** Weighted average spectra of AuMg_8_ nanocluster at 100k and 1000k temperatures.


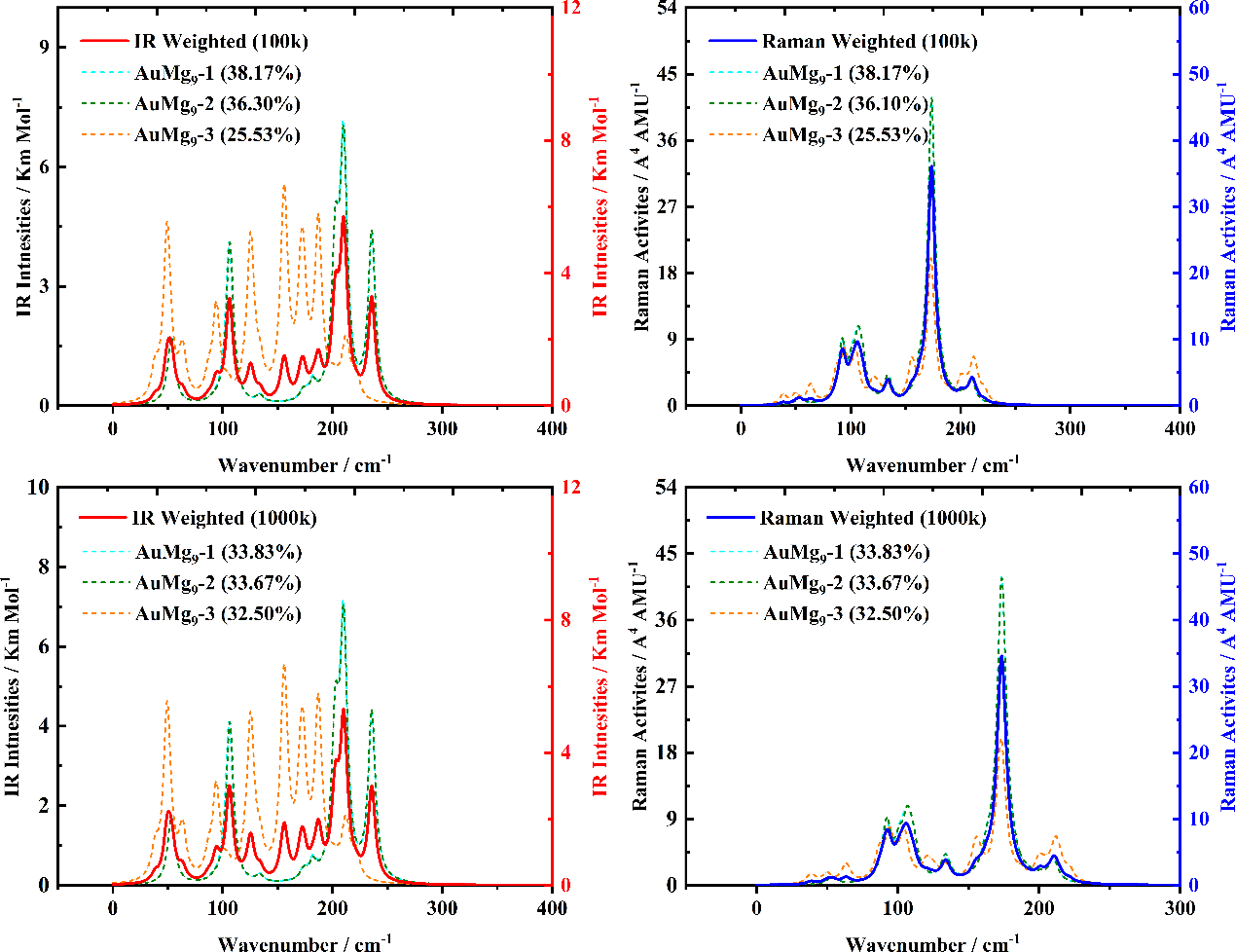


**Figure S2-8.** Weighted average spectra of AuMg_9_ nanocluster at 100k and 1000k temperatures.


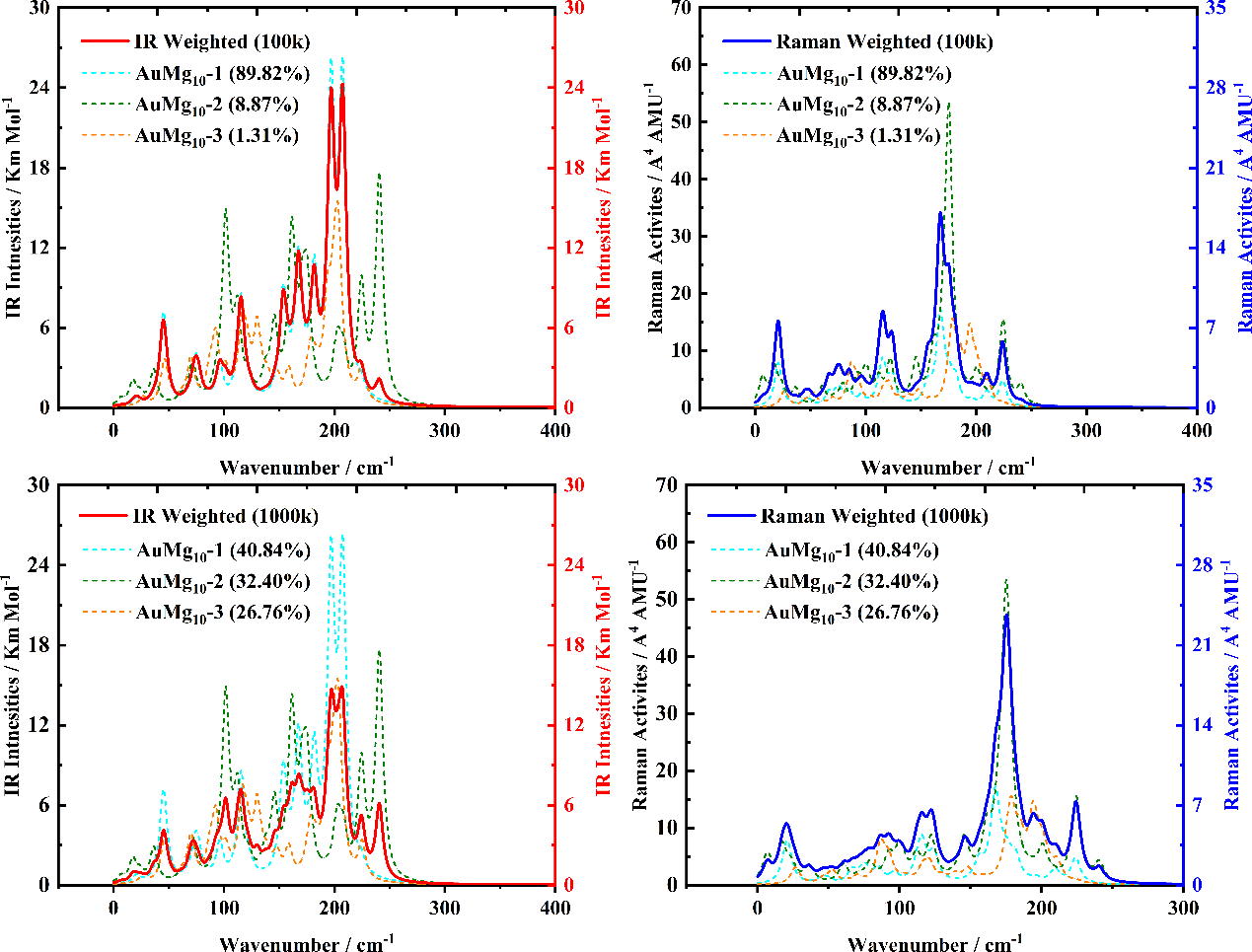


**Figure S2-9.** Weighted average spectra of AuMg_10_ nanocluster at 100k and 1000k temperatures.


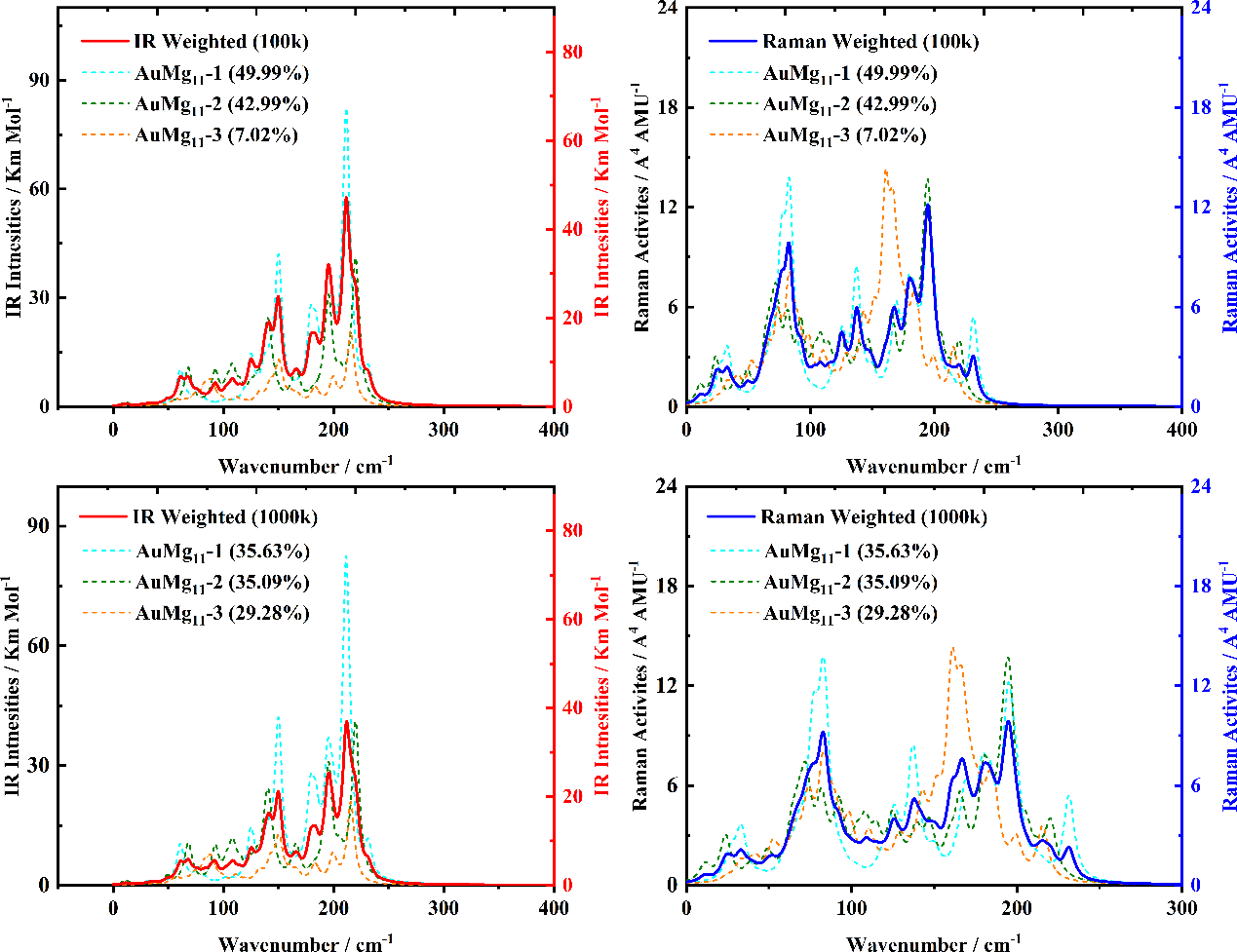


**Figure S2-10.** Weighted average spectra of AuMg_11_ nanocluster at 100k and 1000k temperatures.


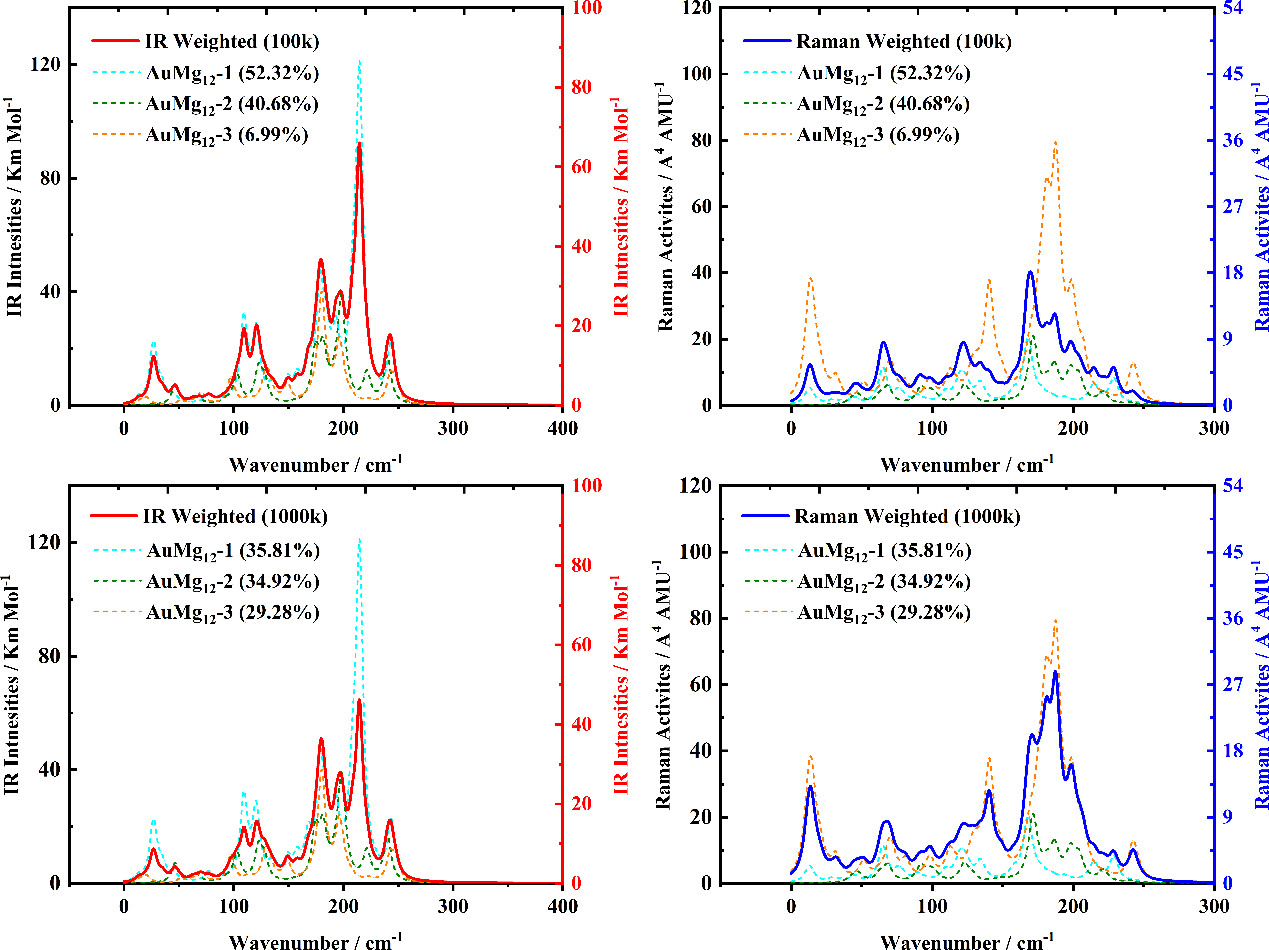


**Figure S2-11.** Weighted average spectra of AuMg_12_ nanocluster at 100k and 1000k temperatures.

Boltzmann distribution

$p_{i}=\frac{n_{i}}{\sum_{j} n_{j}}=\frac{e^{-E_{i}/RT}}{\sum_{j} e^{-E_{j}/RT}}=\frac{Q_{i}}{Q}$ (S3)

*p* is the proportion occupied, *i* is the configuration number, *n_i_* is the number of particles in the *i*th configuration, and E is the energy of the configuration. *T* is the temperature (Kelvin), *R* is the ideal gas constant, and *Q* is called the partition function. In nanocluster studies, the Boltzmann distribution of each isomer can be equivalently written in a form that depends only on the relative energies between the different configurations

$e^{-E_{i}/RT}=e^{-(\Delta E_{i}+E_{\text{Ref}})/RT}=Ce^{-\Delta E_{i}/RT}$ (S4)

$p_{i}=\frac{e^{-\Delta E_{i}/RT}}{\sum_{j} e^{-\Delta E_{j}/RT}}=\frac{Q_{i(\text{Relat})}}{Q_{(\text{Relat})}}$ (S5)

where E_Ref_ represents the lowest energy value (reference value) among all configurations, ΔE is the relative value, and the constant term C corresponding to the reference value is eliminated. The Relat on the Q subscript represents the meaning of Relative. Therefore, to calculate the Boltzmann distribution at different temperatures, it is only necessary to calculate the relative energy of different configurations.
